# Water‐in‐Water Droplets Selectively Uptake Self‐Assembled DNA Nano/Microstructures: a Versatile Method for Purification in DNA Nanotechnology

**DOI:** 10.1002/cbic.202200240

**Published:** 2022-07-01

**Authors:** Marcos K. Masukawa, Yusuke Sato, Fujio Yu, Kanta Tsumoto, Kenichi Yoshikawa, Masahiro Takinoue

**Affiliations:** ^1^ Department of Computer Science Tokyo Institute of Technology 4259 Nagatsuta-cho Midori-ku Yokohama Kanagawa 226-8502 Japan; ^2^ Frontier Research Institute for Interdisciplinary Sciences Tohoku University 6-3 Aramaki-Aza Aoba Aoba-ku Sendai Miyagi 980–8578 (Japan); ^3^ Department of Intelligent and Control Systems Kyushu Institute of Technology 680-4 Kawazu Iizuka Fukuoka 820-8502 Japan; ^4^ Division of Chemistry for Materials Graduate School of Engineering Mie University 1577 Kurimamachiya-cho Tsu Mie 514-8507 Japan; ^5^ Center for Integrative Medicine and Physics Institute for Advanced Study Kyoto University Yoshida Ushinomiya-cho, Sakyo-Ku Kyoto 606-8501 Japan; ^6^ Faculty of Life and Medical Sciences Doshisha University 1-3 Tataramiyakodani Kyotanabe Kyoto 610-0394 Japan

**Keywords:** DNA microtubes, DNA nanotechnology, DNA origami, liquid-liquid phase separation droplets, purification

## Abstract

DNA is an excellent material for constructing self‐assembled nano/microstructures. Owing to the widespread use of DNA as a building block in laboratories and industry, it is desirable to increase the efficiency of all steps involved in producing self‐assembled DNA structures. One of the bottlenecks is the purification required to separate the excess components from the target structures. This paper describes a purification method based on the fractionation by water‐in‐water (W/W) droplets composed of phase‐separated dextran‐rich droplets in a polyethylene glycol (PEG)‐rich continuous phase. The dextran‐rich droplets facilitate the selective uptake of self‐assembled DNA nano/microstructures and allow the separation of the target structure. This study investigates the ability to purify DNA origami, DNA hydrogels, and DNA microtubes. The W/W‐droplet fractionation allows the purification of structures of a broad size spectrum without changes to the protocol. By quantifying the activity of deoxyribozyme‐modified DNA origami after W/W‐droplet purification, this study demonstrates that this method sufficiently preserves the accessibility to the surface of a functional DNA nanostructure. It is considered that the W/W‐droplet fractionation could become one of the standard methods for the purification of self‐assembled DNA nano/microstructures for biomedical and nanotechnology applications owing to its low cost and simplicity.

## Graphical abstract

**Dextran‐rich droplets** display a selective uptake of DNA structures based on size and shape. This property is explored for creating a versatile protocol for the purification of DNA structures used in DNA nanotechnology. The protocol is applicable to a wide size range of DNA structures, with a high yield and short preparation time.

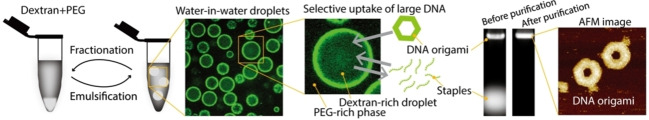

## Introduction

Structural DNA nanotechnology can create both static and dynamic DNA structures in the nanometer to micrometer range in a self‐assembled manner. The self‐assembled DNA nano/microstructures (hereafter referred to as DNA structures) can be designed based on the stability and topology of the hybridized DNA strands,[^1^, ^2^] which achieve the programmability of DNA structures. Because of the programmability, structural DNA nanotechnology has created sophisticated DNA structures such as DNA tiles, DNA origami, DNA tubes, and DNA hydrogels.[^3^, ^4^, ^5^] Such DNA structures have been creatively applied to various functional systems such as molecular robots, plasmonic devices, measuring tools, and scaffolds for nanomaterials.[^6^, ^7^, ^8^, ^9^]

**Figure 1:**
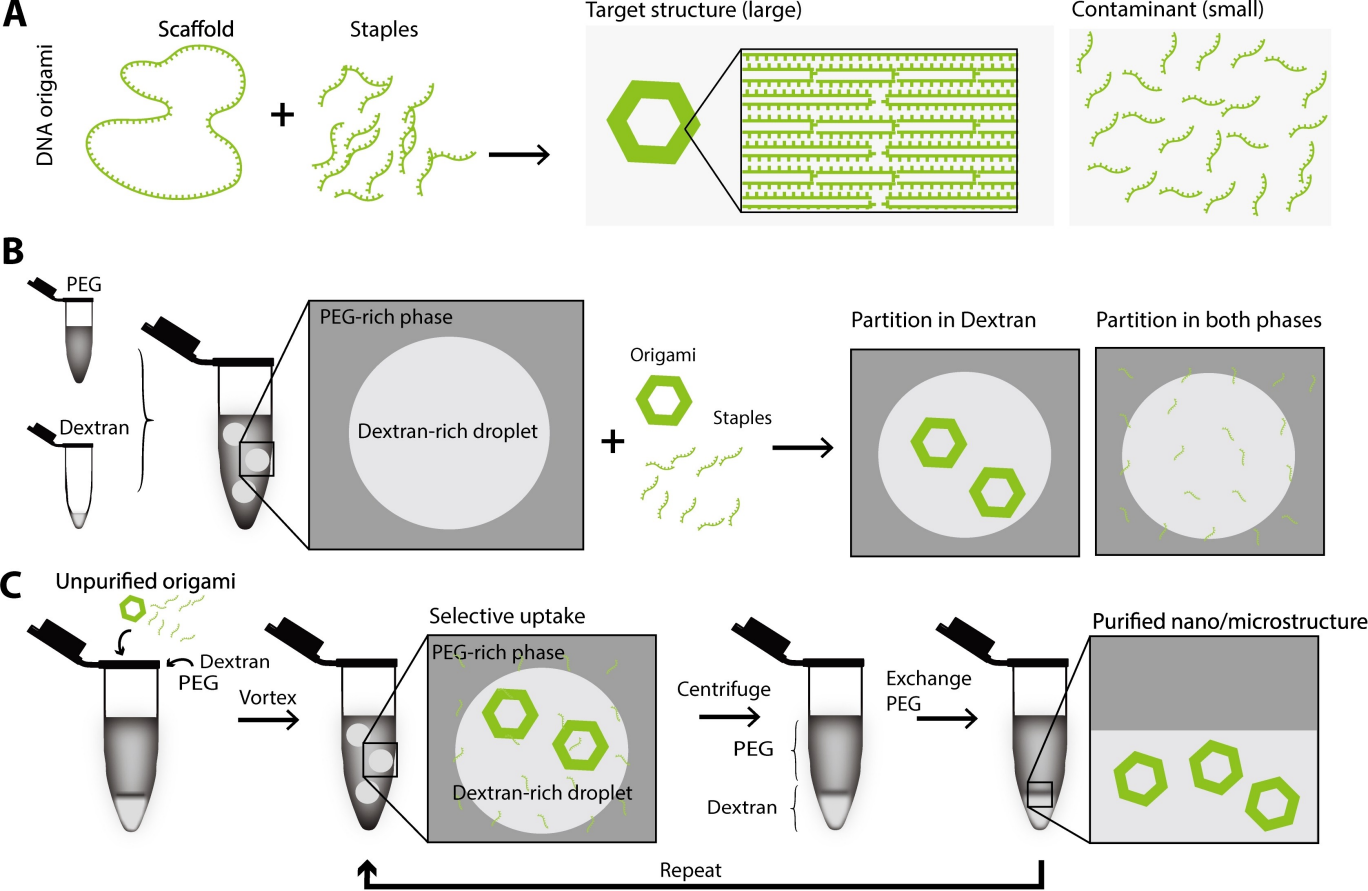
Conceptual illustration of W/W‐droplet purification. (A) DNA origami is formed by hybridization of a single, long‐stranded circular DNA and short ssDNA added in excess. (B) W/W droplets composed of dextran‐rich droplets in a PEG‐rich continuous phase. When unpurified DNA origami is added to the dextran‐rich‐droplet dispersion, large DNA structures partition in the dextran‐rich droplets, while small DNA molecules partition in both dextran‐rich droplets and PEG‐rich phase. (C) W/W‐droplet purification is the process of recovery of the dextran‐rich phase from the dextran‐rich‐droplet in a PEG‐rich phase by centrifugation. Large DNA structures can be concentrated and purified by fractionation and recovery of the dextran‐droplet phase.

Purification is critical for the characterization and application of DNA structures because of the following two reasons. First, such DNA structures are assembled with excess components, typically short single‐stranded DNA (ssDNA) (several tens of nucleotides). For example, DNA origami consists of a long circular ssDNA, called the scaffold, and shorter ssDNA, called staples, that hybridize with the scaffold at specific points and fold it into shape (Figure 1A).^[5]^ If excess components are not removed, they form unintended aggregates and interfere with visualization by fluorescence microscopy, electron microscopy, and atomic force microscopy (AFM). Second, these DNA structures are often modified with functional materials such as fluorophores, proteins, or nanoparticles. The removal of unbound functional materials is necessary to distinguish between the activity of the modified DNA structure and unbound materials.^[10]^ Molecular weight cutoff (MWCO) filtration, gel filtration, and polyethylene glycol (PEG) precipitation have been used to purify DNA structures.[^11^, ^12^] A general limitation of these methods is that they require adaptations to purify DNA structures of different sizes. MWCO filtration (commercial name Amicon™) requires cartridges with different filters for purification of structures of different sizes, whereas gel filtration (commercial name Sephacryl®) requires gel beads of different porosities. PEG precipitation was tested for a large variety of DNA origamis but not for considerably larger or smaller structures, for which adjustments in ionic strength, PEG concentration, or centrifugation time would possibly be required.[^12^, ^13^]

For fractionation and purification of solutes (molecules or nano/microstructures), an aqueous two‐phase system (ATPS) has been used.^[14]^ An ATPS is formed by mixing incompatible aqueous polymers, resulting in phase separation. Generally, solutes added to ATPS preferentially disperse in one of two phases or uniformly disperse in both phases.^[14]^ The partitioning depends on the physicochemical properties of the two aqueous polymers and the added solutes, such as their size, shape, flexibility, and charge. By agitating ATPS, an emulsion‐like suspension composed of water‐in‐water (W/W) droplets in a continuous aqueous phase is formed. In particular, a W/W‐droplet dispersion composed of phase‐separated dextran‐rich droplets dispersed in a PEG‐rich continuous phase has been widely used for DNA purification because of their selective uptake or exclusion of DNA molecules with specific physicochemical properties. The selective uptake of DNA by dextran‐rich droplets was explored for the separation of single‐ and double‐stranded DNAs, high‐ and low‐molecular‐weight DNAs, genomic DNA from a cell lysate, and even chromosomes.[^15^, ^16^, ^17^, ^18^, ^19^] We also observed that genomic DNA and DNA hydrogels were concentrated in the dextran‐rich droplets, whereas short ssDNA were distributed in both dextran‐rich droplets and a PEG‐rich continuous phase.[^20^, ^21^, ^22^] We expected small ssDNA and large DNA structures would have different partitions and be separable by W/W‐droplet fractionation (Figures 1B and 1 C). Therefore, the W/W‐droplet system is expected to be applied to purify DNA structures such as DNA motifs, DNA origami, DNA hydrogel, and DNA microtubes.[^5^, ^23^, ^24^, ^25^]

In this study, we present a versatile purification method of DNA structures over a wide size range based on a W/W‐droplet fractionation. The W/W droplet dispersion for fractionation is composed of dextran‐rich droplets dispersed in a PEG‐rich continuous phase. We investigated the dependence of DNA structure size on the partitioning of DNA structures in dextran‐rich droplets (Figure 1B) and observed the ability to purify DNA structures of a broad size spectrum without changing the protocol. Compared to the previous methods, W/W‐droplet fractionation is fast, effective, and suitable for purifying DNA structures.^[14]^ The size‐dependent selective uptake of DNA by dextran‐rich droplets indicates that W/W droplets, a simple chemical system, can be used to compartmentalize biological molecules in a size‐dependent manner. This study may elucidate the mechanism responsible for the semipermeability of a W/W droplet cooperating with biomacromolecules, leading not only to simpler protocols for the fabrication of DNA structures but also to a better understanding of biological condensates and moreover to the construction of artificial cells.[^25^, ^26^]

## Results and Discussion

### Purification of DNA origami with W/W‐droplet fractionation

First, we attempted to purify DNA origami using W/W‐droplet fractionation. The DNA origami tested was a two‐dimensional hexagonal DNA origami (Figure 1A, Supporting Materials and Methods 1.1, Figure S1, Table S1).^[27]^ Purification based on W/W‐droplet fractionation was performed by centrifuging the dextran‐rich‐droplet dispersion, replacing the supernatant PEG‐rich phase, and re‐emulsifying the dextran‐rich droplets in a PEG‐rich phase three times (Figure 1C). The dextran‐rich droplets contained 8.33 wt% PEG 6 K (molecular weight 6,000 g mol^−1^) and 0.833 wt% dextran 200 K (molecular weight 200,000 g mol^−1^) with 1×tris‐acetate‐ethylenediaminetetraacetic‐acid (TAE) buffer and 5 mM magnesium acetate. Under this condition, dextran‐rich droplets were formed in the PEG‐rich continuous phase. We then added either DNA origami staples or unpurified DNA origami to the dextran‐rich‐droplet dispersion and observed their partitions.

**Figure 2:**
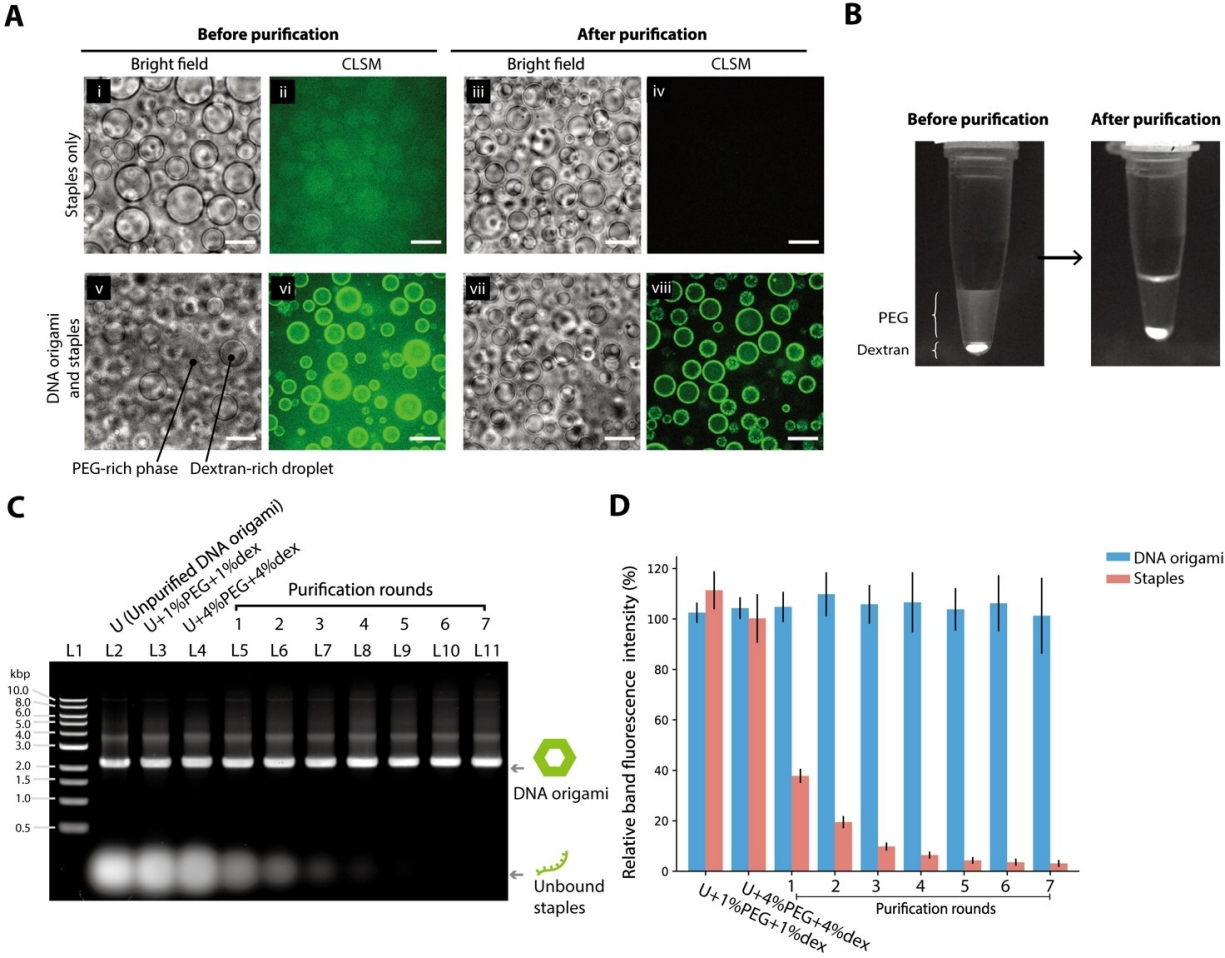
Partitioning of DNA origami components in dextran‐rich droplets allows the separation of DNA origami and excess staples. (A) Bright field images (i), (iii), (v), and (vii) and CLSM images (ii), (iv), (vi), and (viii) of dextran‐rich droplets containing fluorescently stained DNA before and after purification by W/W‐droplet fractionation. The top row shows a sample containing only the staples and the bottom row shows a sample containing unpurified DNA origami (mixture of folded DNA origamis and unbound staples). Scale bars: 20 μm. (B) Transillumination fluorescence photograph of the completely phase‐separated dextran and PEG phases after the centrifugation of the dextran‐rich‐droplet dispersion before and after the purification, indicating PEG‐rich phase (top) and dextran‐rich phase (bottom). (C) Agarose gel electrophoresis of unpurified DNA origami only (L2), with PEG 1 wt%+dextran 1 wt% (L3), with PEG 4 wt%+dextran 4 wt% (L4), and after W/W‐droplet fractionation by one to seven fractionation rounds (L5–L11). L1: 1 kbp ladder marker. (D) Quantification of the relative intensity of the agarose gel electrophoresis bands, indicating the intensity of the band associated with the folded DNA origamis and the unbound excess staples.

Figure 2A shows the microscopy images of dextran‐rich‐droplet dispersion containing DNA before and after the purification. The panels (i), (iii), (v), and (vii) in Figure 2A are bright‐field microscope images showing the dextran‐rich droplets, while the panels (ii), (iv), (vi), and (viii) are the fluorescence images obtained using a confocal laser‐scanning microscope (CLSM), showing the location of DNA origami and the excess staple DNAs fluorescently stained with SYBR Gold. Figure 2A(ii), which depicts the condition of only the staple DNAs, shows that the staples were partitioned on both the dextran‐rich droplets and the PEG‐rich phase, with a slight preference for the dextran‐rich droplets. Therefore, the staple DNAs were almost completely removed after purification by three rounds of W/W‐droplet fractionation (Figure 2A(iv)). When DNA origamis and the excess staple DNAs before purification were added to the dextran‐rich‐droplet dispersion (Figure 2A(vi)), the fluorescence was concentrated in the dextran‐rich droplets. The background fluorescence in Figure 2A(vi) is attributed to the unbound staples. After purification by three rounds of W/W‐droplet fractionation, the background fluorescence was removed (Figure 2A (viii)). Thus, we found that the DNA origamis were preferentially partitioned into the dextran‐rich droplets and that the hexagonal DNA origami (whose diameter and thickness were ∼100 nm and ∼2 nm, respectively) could be separated from the staples ∼5 nm in hydrodynamic diameter) by W/W‐droplet fractionation. One possible hypothesis^[28]^ for DNA accumulation in the dextran‐rich droplets is that a double‐stranded DNA, a semiflexible chain, can penetrate into the nanoscale cavities of a dextran‐rich phase in contrast to a PEG‐rich phase, which is completely filled with flexible PEG chains. On the other hand, a short single‐stranded DNA has no preference for a phase containing either PEG or dextran due to its small size. We also observed the preferential accumulation of DNA origami at the circumference area in the dextran‐rich droplets. Because this phenomenon is out of scope in this work, we here focus on the preferential partitioning into the dextran‐rich droplets and purification of DNA structures and will explore this phenomenon in a future study.

The separation of DNA origami from the staples was also visualized with the naked eye by transilluminating an ATPS in a test tube before and after purification (Figure 2B). Before purification, higher fluorescence intensity was observed in the dextran‐rich phase, with background fluorescence in the PEG‐rich phase. After purification, the fluorescence in the PEG‐rich phase was almost removed, while that in the dextran‐rich phase remained.

We analyzed the DNA origami after W/W‐droplet purification by a different number of fractionation rounds using agarose gel electrophoresis (Figure 2C). In agarose gel electrophoresis, the staples exhibited higher electrophoretic mobility and appeared as a diffuse band at the bottom of the gel, whereas the DNA origami appeared as a sharp upper band, as shown in Figure 2C (Figure 2C, Lane L2). Lanes L5–L11 in Figure 2C show samples after purification by one to seven rounds of fractionation, respectively. We observed that the staples were progressively removed, while the DNA origami was retained at each fractionation round.

To estimate the purification yield, we tested the effect of PEG and dextran residues on the migration and fluorescence intensity of DNA in the agarose gel (Figure 2C, Lanes L3–L4). We added 1 wt% PEG and 1 wt% dextran, or 4 wt% PEG and 4 wt% dextran, to the unpurified DNA origami (Figure 2C, Lanes L3–L4). We observed no effect of PEG and dextran on the electrophoretic mobility of DNA, although their residues slightly increased the fluorescence intensity of the SYBR Gold used to stain DNAs, requiring an appropriate reference for quantification of nucleic acids. Therefore, the purification yield of Lanes L5–L11 in Figure 2C was estimated based on their relative band fluorescence intensity compared to the fluorescence intensity of the unpurified DNA origami (Figure 2C, Lane L2) as shown in Figure 2D: the relative band fluorescence intensity was determined to be close to 100 % regardless of the number of fractionation rounds (see also Supporting results 3.2, Figure S3), suggesting the high yield of purification. This result shows that three or more fractionation rounds were required to reduce the unbound staples to less than 10 % of the initial amount.

Purification by W/W‐droplet fractionation was advantageous in terms of the purification yield for a small sample volume (5 μL of 15 nM DNA origami). MWCO filtration and gel filtration resulted in higher losses of DNA origami when the condition was not optimized (Supporting Materials and Methods 1.2, Figure S4) because of the adsorption of DNA origami on the filter surface, although the yield was brought close to 100 % by pre‐coating of the filter with proteins or surfactants^[10]^ or modifying the buffer.^[29]^ With W/W‐droplet purification, there is no solid matrix responsible for overhead losses, and purification can be performed without further optimization. In addition, W/W‐droplet purification can be performed in approximately 5 min, whereas MWCO filtration, gel filtration, and PEG precipitation require more than 30 min.

### Investigation of activity of a functional DNA origami after purification

**Figure 3:**
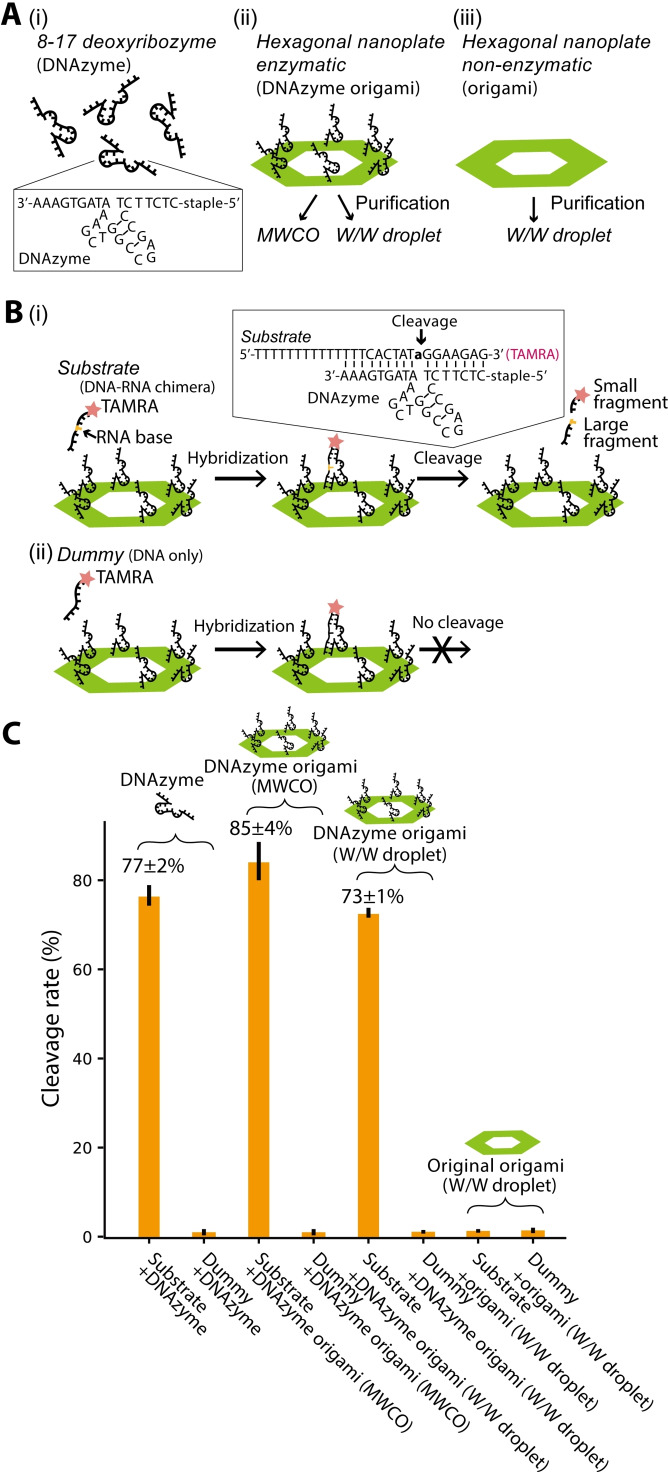
Investigation of the effect of W/W‐droplet purification on DNA catalytic function. (A) Comparison of the catalytic activity of free DNAzyme (i), DNAzyme origami (ii) purified by either MWCO filtration or W/W‐droplet fractionation, and original DNA origami without DNAzyme (iii) purified by W/W‐droplet fractionation. (B) (i) The substrate of the DNAzyme is a DNA‐RNA chimera. The DNAzyme cleaves the molecule adjacent to the RNA base (colored with yellow), producing smaller fragments. (ii) A pure DNA molecule with the sequence (′Dummy′) is not cleaved. (C) Cleavage rate measured by the native PAGE.

Although the W/W‐droplet purification is rapid and high‐yielding, the contamination of the residual polymers is a potential drawback. The polymers can be removed by further centrifugation, precipitation, buffer exchange columns, chromatography, or electrophoresis, but a second purification procedure is disadvantageous in terms of practicality.^[16]^ Therefore, we investigated whether the polymers remaining after W/W‐droplet purification could affect the ability of DNA structures to interact with other molecules. We prepared a hexagonal DNA origami modified with 8–17 deoxyribozymes (hereafter, DNAzymes) (Figure 3A).^[6]^ In this design, twelve staples of the original hexagonal DNA origami were replaced by modified staples containing the DNAzyme at the 3’ end (Figure 3A, see Supporting Information Materials and Methods 1.1, Table S2, Figure S5 for more details on the structure design). The DNAzyme can cleave a single‐stranded DNA‐RNA chimera (referred to as a substrate) at its RNA base (Figure 3B), whereas a pure ssDNA with the same sequence could not be cleaved (referred to as a dummy; serving as a negative control). The substrate was fluorescently labeled so that the reaction could be followed by the change in the size of the fluorescent molecule (Figure 3B) in a native polyacrylamide gel electrophoresis (PAGE) experiment.

Figure 3C shows the cleavage ratio of the substrate by the DNAzyme‐modified DNA origamis (hereafter referred to as DNAzyme origamis). We compared the activity of free DNAzymes, DNAzyme origami purified by MWCO filtration, DNAzyme origami purified by W/W‐droplet fractionation, and the original DNA origami without DNAzymes. The enzymatic reaction of DNAzyme was performed by annealing the substrate (or dummy) with DNAzyme origami (or free DNAzyme, or original DNA origami) and cooling the solution temperature from 40 °C to 25 °C at a rate of −0.5 °C min^−1^. Immediately after the annealing, we performed the native PAGE and then evaluated the percentage of the cleaved substrate by image analysis of the native PAGE result (Figure 3C; see Supporting results 3.3, Figure S6). Both DNAzyme origami cleaved approximately the same amount of substrate as the free DNAzyme. The cleavage of the negative control was negligible. Thus, it was confirmed that the surface accessibility of the DNA origami was sufficiently preserved even after W/W droplet purification, which is important for the function of DNA structures.

### Theoretical investigation of the purification of DNA origami with W/W‐droplet fractionation

Next, we numerically investigated the dependence of the purification efficiency on the DNA structure size. The theoretical details are described in the Supporting Theoretical Notes with Figure S2 in the Supporting Information using data and theories obtained from the literature.[^8^, ^14^, ^30^, ^31^, ^32^, ^35^] The purification efficiency (E ) is defined as follows:

(1) $$E=\frac{Y_{\mathrm{target}}^{n}-Y_{\mathrm{contaminant}}^{n}}{Y_{\mathrm{target}}^{n}+Y_{\mathrm{contaminant}}^{n}}$$

where $Y_{\mathrm{target}}^{n}$ and $Y_{\mathrm{contaminant}}^{n}$ are the yields of the target structures and contaminant molecules after the purification by n rounds of fractionation, respectively.

**Figure 4:**
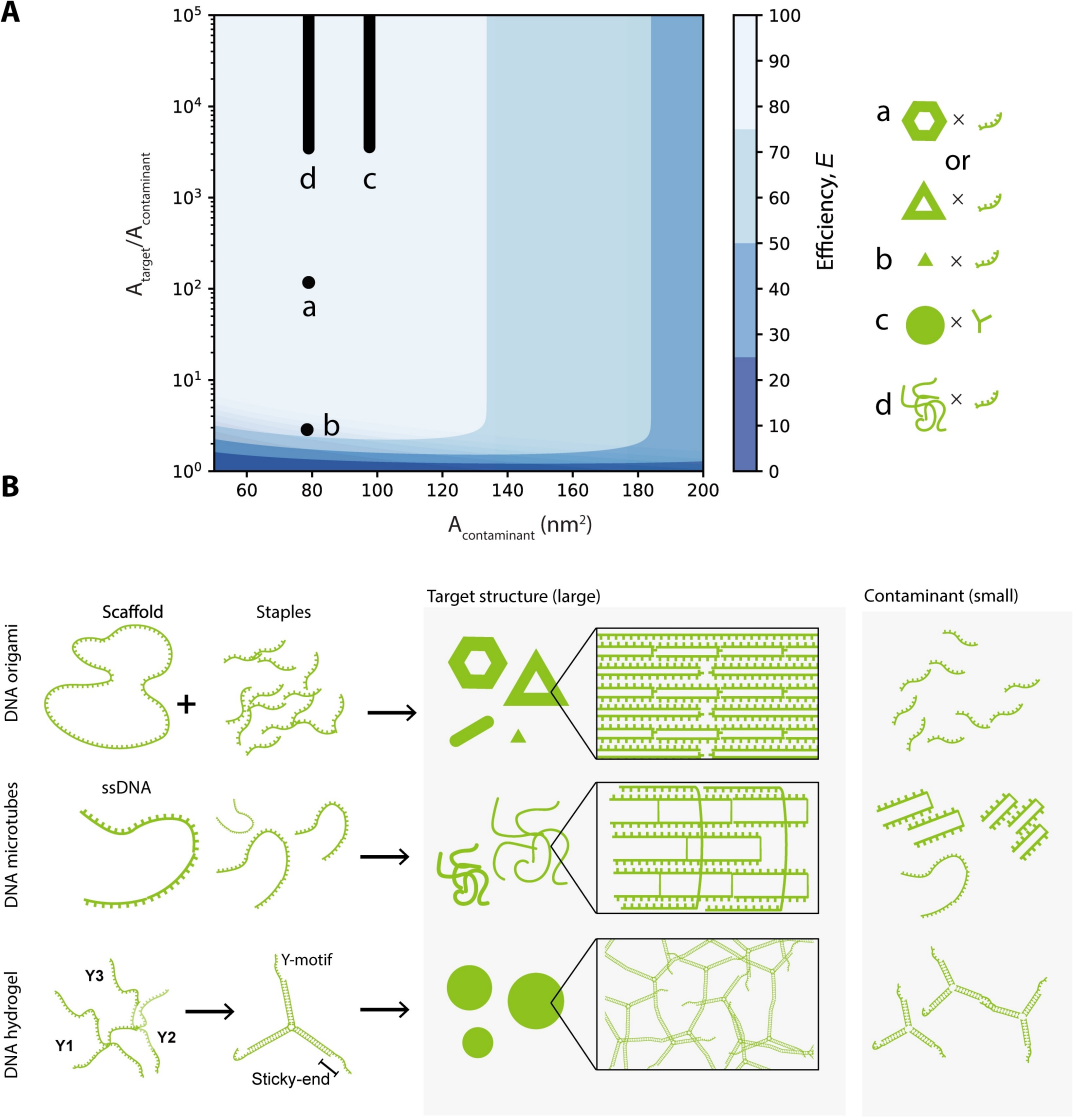
Theoretical investigation of the purification efficiency. (A) Separation efficiency of a mixture of target structures and contaminant molecules. The mixture contains a target structure with surface area $A_{target}$ and a contaminant molecule with surface area $A_{contaminant}$ . The plot represents the purification efficiency according to Equation 1, given the surface area of the smaller contaminant molecule ${(A}_{contaminant}$ ) and the ratio of the surface areas $\left(A_{target}/A_{contaminant}\right)$ . Point (a) represents the separation of hexagonal or triangular DNA origami and unbound staples; point (b) represents the separation of mini‐triangular DNA origami and unbound staples; line (c) represents the separation of DNA hydrogels and unbound Y‐motifs; line (d) represents the separation of DNA microtubes and unbound staples (see Supporting materials and methods 3.5, calculated with the aid of equations and data from the previous studies[^30^, ^31^, ^32^, ^34^, ^35^]). (B) DNA self‐assembly processes that generate large target structures and smaller contaminants.

Figure 4A shows the separation efficiency of a pair of target structures and contaminant molecules after three rounds of W/W‐droplet purification. Efficient separation occurred only within a range of sizes; that is, the efficiency is higher for small contaminant molecules and large target structures, as expected (the left upper area of Figure 4A). In contrast, when the contaminant molecule is small and the target structure has a similar size (the bottom left area of Figure 4A), the efficiency is low because both are removed by the purification. When the contaminant molecule is large (the right area of Figure 4A), the efficiency is low because the contaminant molecule is retained after purification.

Point (a) in Figure 4A shows the expected purification efficiency of separation of the hexagonal DNA origami experimentally investigated in Figure 2 ∼100 nm in diameter and ∼2 nm in thickness) from the staple molecules ∼5 nm in hydrodynamic diameter). The theoretically estimated efficiency qualitatively matches the experimental results.

To apply this purification method to various DNA structures, we numerically investigated various DNA structures in Figure 4B using Equation (1). Because the triangular DNA origami^[5]^ has approximately the same size as the hexagonal DNA origami, the expected efficiency is plotted at point (a). Point (b) shows the expected efficiency for a mini‐triangular DNA origami (∼15 nm in diameter and ∼2 nm in thickness), which is formed from a small scaffold called M1.3, whose size is one‐tenth that of the M13mp18 scaffold.^[33]^ Lines (c) and (d) in Figure 4A depict the expected efficiencies of purification of DNA hydrogels and DNA microtubes, respectively, which are marked by a line because these DNA structures lack a defined size, and range from hundreds of nanometers to several micrometers. Therefore, the mini‐triangular DNA origami is probably the smallest structure that can be purified using W/W‐droplet fractionation in this condition.

### W/W‐droplet purification applied to a wide range of DNA structures

**Figure 5:**
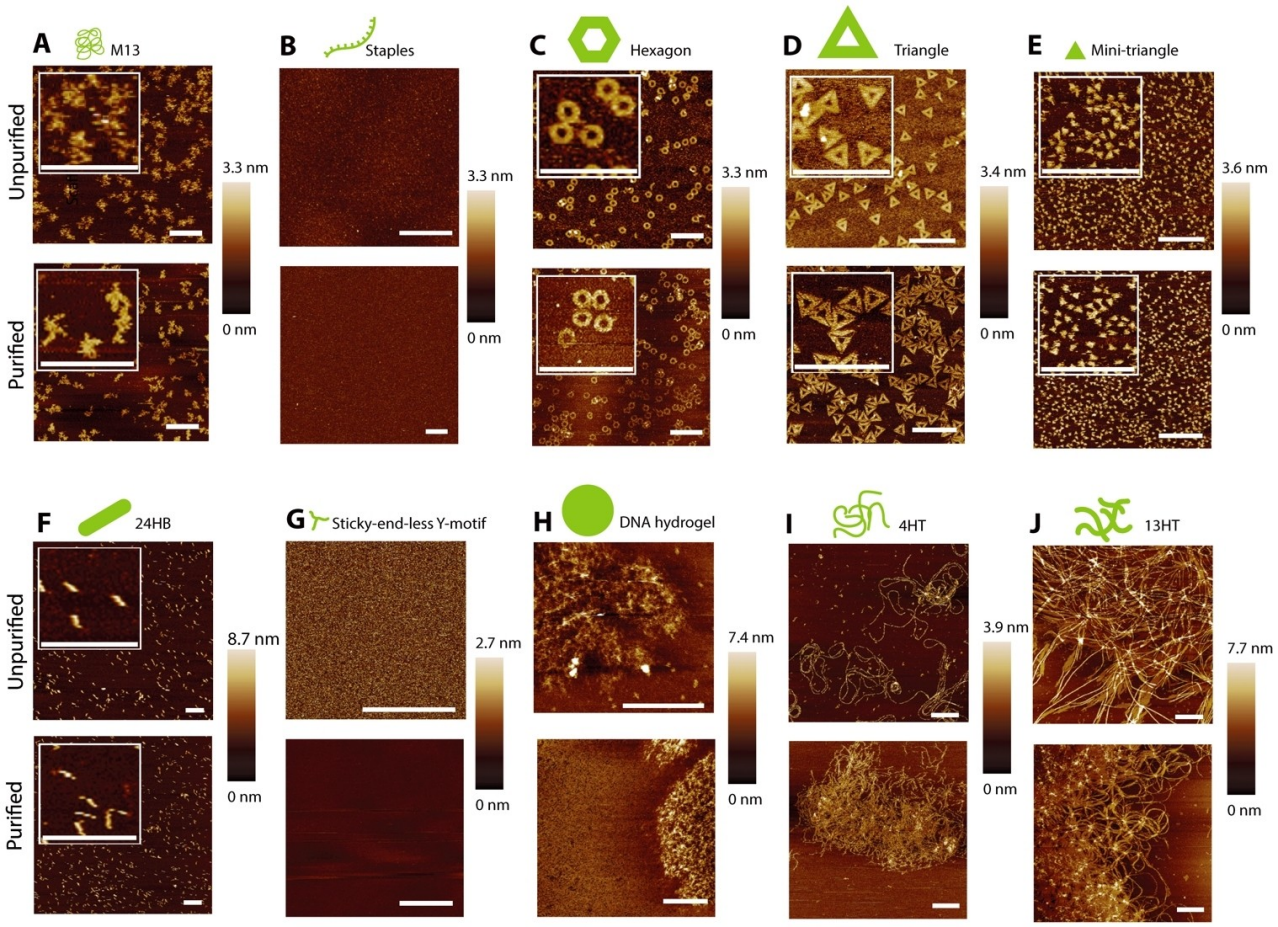
AFM image of various DNA structures, unpurified and purified by three rounds of W/W‐droplet fractionation. (A) M13 scaffold. (B) Staples. (C) Hexagonal DNA origami. (D) Standard‐size triangular DNA origami. (E) Mini‐triangular DNA origami built from the M1.3 scaffold. (F) 24HB. (G) Sticky‐end‐less Y‐motif. (H) DNA hydrogel. (I) 4HT. (J) 13HT. Details of the DNA structures can be found in the Supporting Information Table S3. Scale bars: 500 nm.

**Figure 6:**
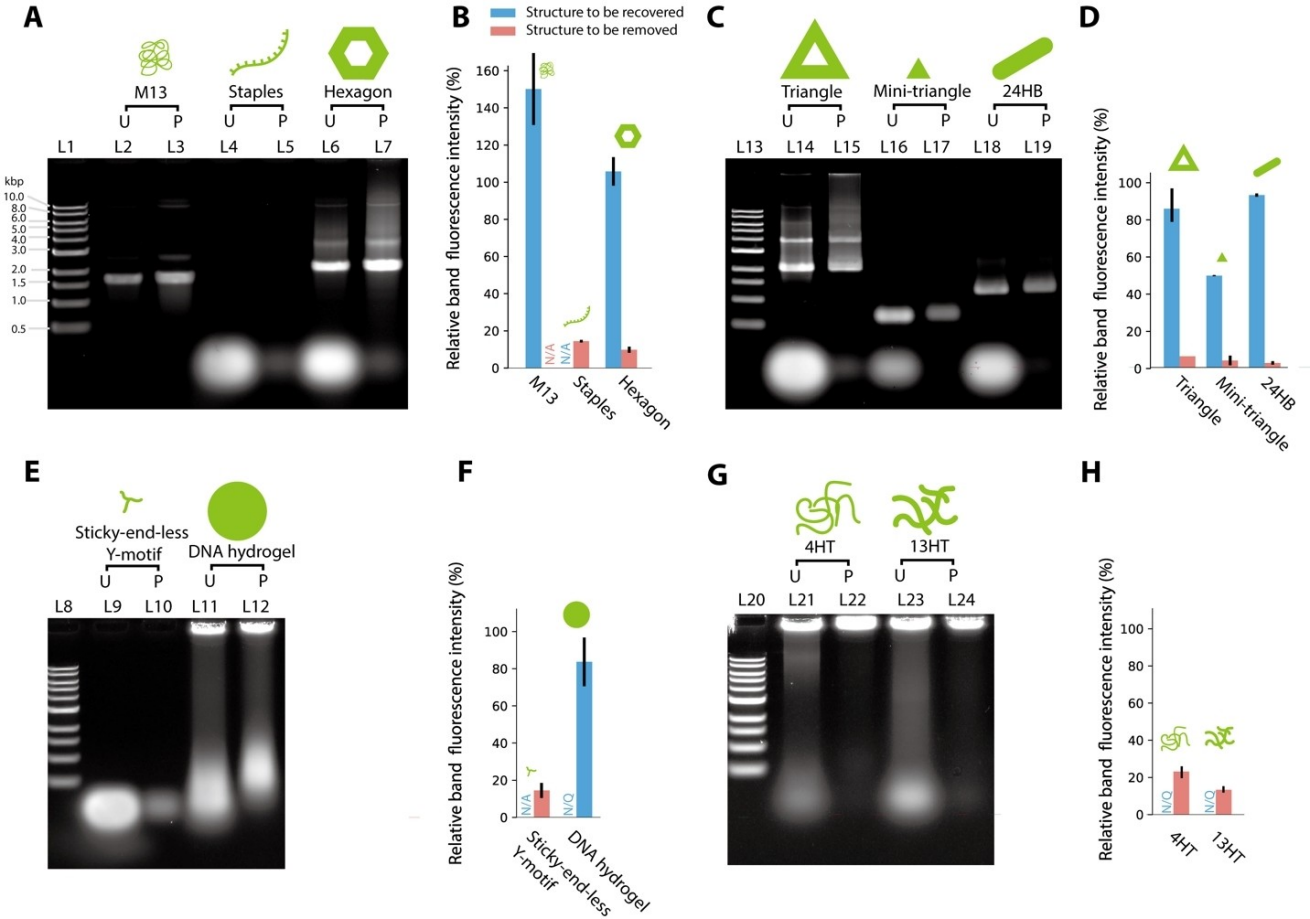
Agarose gel electrophoresis of various DNA structures before and after purification by W/W‐droplet fractionation. (A) Hexagonal DNA origami, M13 scaffold, and staples. (B) Quantification of (A). (C) DNA origami with different sizes and shapes: standard‐sized triangle, mini‐triangle, and 24HB. (D) Quantification of (C). (E) DNA Y‐motifs and DNA hydrogels. (F) Quantification of (E). (G) DNA microtubes: 4HT and 13HT. (H) Quantification of (G). L1, L13, L8, L20: 1 kbp ladder marker. ‘U’ indicates ‘unpurified’. ‘P’ indicates ‘purified’. ‘N/A’ indicates ‘not applicable’. The large DNA structures that were clogged in the wells of the agarose gel (DNA hydrogel, 4HT and 13HT microtubes) were not quantified. ‘N/Q’ indicates ‘not quantified’.

To investigate the theoretical expectations, we experimentally tested the purification of various DNA structures with a wide range of sizes and shapes, as shown in Figure 4B (see Supporting Materials and Methods 1.3, Supporting Table S3), and investigated them by AFM (Figure 5) and agarose gel electrophoresis (Figure 6, Figure S7) before and after purification by the three rounds of W/W‐droplet fractionation.

Figure 5A and L2–L3 of Figure 6A show that the M13 scaffold was recovered by W/W‐droplet purification. Figure 5B and L4–L5 of Figure 6A show that the staples were removed by the purification as expected. In the AFM image (Figure 5B), the staples appear as small aggregates, which were observed in the unpurified sample, but not in the purified sample. Figure 5C and L6–L7 of Figure 6A suggest that the hexagonal DNA origami was purified with a high yield, which is consistent with the results shown in Figure 2. In addition, Figure 5C demonstrates that the purification maintained its structural integrity. Agarose gel electrophoresis results for the scaffold, staples, and hexagonal DNA origami are summarized in Figure 6B. Although the relative band fluorescence intensity of M13 after purification (Figure 6B) was higher than 100 %, that is because the presence of PEG and dextran increases the fluorescence of SYBR Gold, as shown in Figure 2C.

Second, we tested DNA origamis with different sizes and shapes: a standard‐sized triangular shape (∼100 nm), a mini‐triangular shape (∼15 nm), and a cylindrical shape comprising of twenty‐four helix bundles (24HB) (∼80 nm) (Table S3).[^24^, ^31^] The AFM images are shown in Figures 5D–5F; the agarose gel electrophoresis results and the relative band fluorescence intensity of purification are shown in Figures 6C and 6D, respectively. The relative band fluorescence intensities of the standard‐sized triangular DNA origami and 24HB DNA origami were (87.3± 9)% and (92.7±0.9)%, respectively (Figure 6C, L14–L15 and L18–L19; Figure 6D), while their structural integrity was maintained (Figures 5D and 5F). The results suggest a high yield of purification even for these DNA structures. Their purification efficiencies E were (86.6±1.6)% for the triangular DNA origami and (94.8±1.9)% for the 24HB DNA origami, which were calculated using Equation (1) by assuming the relative band fluorescence intensity to be the yield. The mini‐triangular DNA origami could also be purified, but with a lower relative band fluorescence intensity of (49.4±3.7)% and efficiency E of (73.7±0.2)% (Figures 5E; Figure 6C, L16–L17; Figure 6D), suggesting a lower yield, as theoretically predicted (see Figure 4A, point (d)).

Third, we have attempted to purify motif‐based DNA structures. These structures are based on a Y‐shaped motif comprising three ssDNAs (Table S4). One of the structures, the sticky‐end‐less Y‐motif, was non‐interacting and remained in solution, whereas those with sticky ends formed microscopic aggregates called DNA hydrogels. Similar to the staples, the DNA hydrogels without sticky ends were observed as small aggregates in AFM (Figure 5G) and were mostly removed by W/W‐droplet purification (Figure 6E, L9‐10). The DNA hydrogels were recovered after purification, but smeared bands were observed during electrophoresis (Figures 5H; Figure 6E, L11–L12) before and after purification. This is probably because the Y‐motifs form a reversible network.^[23]^ Thus, when the smaller aggregates were removed from the solution, the Y‐motifs dissociated from the larger DNA structure. These results suggest that the purification itself is not applicable to motif‐based DNA hydrogels, although the Y‐motifs can be removed by the W/W‐droplet purification. The agarose gel electrophoresis results for sticky‐end‐less Y‐motifs and DNA hydrogel are summarized in Figure 6F.

Finally, we examined the purification of DNA microtubes 4HT and 13HT, which were formed from DNA tile bundles.^[24]^ These tubes have a definite diameter but an undefined length that can span several micrometers. The 4HT and 13HT were recovered after purification, as shown in the AFM images (Figures 5I and 5 J). Agarose gel electrophoresis (Figure 6G, L21–24) shows that smaller aggregates were successfully removed from the samples. These results indicated that the W/W‐droplet purification method is suitable for micrometric structures based on DNA tiles, as we expected based on their size (Figure 4A, line (a)). The agarose gel electrophoresis results for the 4HT and 13HT microtubes are summarized in Figure 6H.

These results indicate that W/W‐droplet fractionation can purify DNA structures across a broad range of sizes without changing the protocol and is particularly useful for purifying DNA origamis and DNA tiles.

## Conclusion

Purification by W/W‐droplet fractionation has the advantages of time and versatility. First, during W/W‐droplet fractionation, the macroscopic phases and the molecules they contain can be separated in a few seconds under centrifugation instead of minutes required to separate molecules in a single‐phase system using glycerol gradients, polymer precipitates, or solid matrices (such as MWCO and gel filtration). Second, W/W‐droplet fractionation does not require any changes to the protocol for purifying DNA structures of different sizes. We have shown that the W/W droplets can be used to purify DNA structures with a broad size spectrum, including DNA origami with various scaffolds and DNA tile‐based microtubes, while maintaining their structural integrity. Our purification method may be extended by combining other effects such as physicochemical properties of other molecules or nanoparticles with the size effect of DNA structures. Moreover, optimization of conditions, such as polymer concentration, polymer length and type, buffer conditions, and ionic strength, may allow this method to be used for purification in more difficult separation conditions.

Although the dextran and PEG residues remained in the solution during W/W‐droplet purification, the presence of the polymers did not significantly affect the activity of DNA origami modified with DNAzymes. As noted by Albertsson, the presence of polymers after W/W‐droplet purification is usually a beneficial aspect and leads to the increased stability of proteins and nucleic acids.^[14]^

Therefore, W/W‐droplet fractionation offers advantages over the other methods currently used in structural DNA nanotechnology. It is the most suitable when large DNA structures need to be separated from small contaminant molecules, when costs and time are limited, when sample volumes and concentrations are low, and when polymer contamination is not detrimental to the application. Under these conditions, we believe that the W/W‐droplet purification methods have great potential in preparing DNA structures at laboratory and industrial scales.

## Experimental Section

**Purification by W/W‐droplet fractionation**: The aqueous two‐phase system used to purify the samples consisted of 8.33 wt% PEG 6 K (Wako, 169‐09125, lot. PTL1562), 0.833 wt% dextran 200 K (Wako, 041‐22612, lot. WDE0888), 1×TAE buffer (tris base 40 mM, acetic acid 20 mM, ethylenediaminetetraacetic acid (EDTA) 1 mM, pH 8.3, Nacalai Tesque 35430‐61, lot L9P0626), and 5 mM magnesium acetate tetrahydrate (99.9 % purity, Wako, 33‐10012, lot KLN3877). For most purification experiments, 5 μL of the sample to be purified was added to 40 μL of the dextran‐rich‐droplet dispersion. For each fractionation round, the solution was shaken for 30 s and then centrifuged at 1620×g for 1 min in a benchtop centrifuge (Waken Puchimaru, model 2320). After centrifugation, some supernatant was removed, leaving a volume $f_{n}$ at the bottom of the tube (n= the $n^{th}$ fractionation rounds). The sample was then replenished to 40 μL with a fresh PEG‐rich phase (PEG 8.33 wt%, 5 mM magnesium acetate, 1×TAE buffer). Typically, $f_{1},f_{2},f_{3}$ were 15, 15, and 5 μL, respectively. For most experiments, three rounds of W/W‐droplet purification were performed. Purification experiments were performed with slight modifications. Triangular, mini‐triangular, and 24HB DNA origami were purified using 2 μL of a sample. DNA hydrogel, sticky‐end‐less Y‐motif, 4HT and 13HT microtubes were purified using 3 μL of the sample. To compare the relative band fluorescence intensity by agarose gel electrophoresis, 20 % of the initial sample was compared with 20 % of the volume obtained after purification.

**Design and preparation of the hexagonal DNA origami**: See Supporting Materials and Methods 1.1 for more details on the hexagonal DNA origami design. The two‐dimensional DNA origami was prepared by annealing 15 nM M13mp18 DNA (Tilibit Nanosystems type p7249 lot M1‐1‐5), and 105 nM of the staples from Table S1 purchased from Eurofins Genomics (salt‐free grade) in folding buffer (1×TAE buffer containing 10 mM magnesium acetate). A PCR machine (Eppendorf Flexlid, Mastercycler, Nexus X2) was used for thermal annealing. The sample was heated to 95 °C and then cooled to 25 °C at a rate of −1 °C min^−1^.

**Scaffold and staples**: The scaffold‐only control consisted of 15 nM M13mp18 DNA in 1×TAE buffer containing 10 mM magnesium acetate. The staples‐only sample comprised 105 nM staples for the hexagonal DNA origami in 1×TAE buffer containing 10 mM magnesium acetate.

**Other purification methods (MWCO and gel filtration)** : Amicon ultra filters 0.5 mL, 30 kDa (Merck Millipore) were used for MWCO filtration. Sephacryl S‐300 HR (Sigma Aldrich, 65546–95‐4) was used for gel filtration. See Supporting Materials and Methods 1.2 for details.

**Various DNA structures**: See Supporting Materials and Methods 1.3 for more details.

**Microscopy observation**: The bright field and CLSM images were captured by an inverted scanning microscope IX81 (Olympus Corporation) equipped with a spinning‐disk confocal system (Yokogawa CSU−X1) 488 nm laser, Coherent Obis) and an EM CCD camera (Andor and iXon X3). We collected the images with software Andor iQ v.3.6.2 and analyzed them using ImageJ[^36^, ^37^] software, uniformly corrected for brightness and contrast.

**Agarose gel electrophoresis**: A 1×TAE buffer with 5 mM magnesium acetate was used to cast a gel of 1 wt% agarose S (Wako Chemical 312‐001193, lot. 20038B). Samples were diluted six‐fold in a buffer containing loading dye xylene cyanol 0.016 wt% and glycerol 6.6 wt%. The agarose gel was run at 110 mA for 50 min at a constant current in the same buffer used to cast the gel. After the samples were run, the gel was immersed in a solution of 0.1 μL mL^−1^ SYBR Gold (Invitrogen, S11494 lot. 2174893) in 1×TBE buffer (pH 8.3, Nippon Gene, 318–90041, lot 01761 A) for 30 min, washed in Milli‐Q water, and imaged using a FLA5000 fluorescence image analyzer (Fuji Film) with an excitation wavelength of 473 nm and the LPG filter. To analyze the relative DNA concentration of each band in the agarose gel electrophoresis gel, the intensity profile of the band was measured using ImageJ[^36^, ^37^] and exported in tabular form. A custom Python script was used to measure the DNA concentration in each band. In the script, the band region was manually selected, and the asymmetric least‐squares smoothing algorithm was used to remove the baseline associated with the background intensity. The area of the curve minus the baseline was then integrated using the trapezoidal algorithm.

**AFM imaging**: A 5 mm×5 mm piece of mica (Alliance Biosystems, Muscovite mica grade V‐1 01872‐ CA, lot. 1190919) was mounted on a metal holder using melted wax. A piece of mica was then cleaved with tape, and 1 μL of a sample was added to the mica and incubated for 1 min. Folding buffer (10 μL) was added. The samples were observed in solution using a Bruker Nanoscope V multimode 8 AFM with a Nanoscope 8.15, Scanasyst software in fluid mode using a Scanasyst fluid cantilever probe. Images were analyzed using Nanoscope analysis software v. 1.4. We used the flatten and clear functions and then subtracted a linear baseline from the images.

**Measurement of the catalytic activity of DNAzyme origami**: The activity of 8–17 deoxirybozyme (DNAzyme) was tested. The DNAzyme was either present in free form as 5’‐[AminoC6]TTATTATTATCTCTTCTCCGAGCCGGTCGAAATAGTGAAAA‐3’ (Operon, HLPC purification) or was incorporated into the hexagonal DNA origami to form the structure referred to as DNAzyme origami (see Supporting Materials and Methods 1.1, Supporting Results 3.3). The DNAzyme was active on the substrate but inactive on the dummy molecule, both obtained from Hokkaido System Science (HLPC purification). The DNAzyme staples used in the DNAzyme origami were purchased from Eurofins Genomics (OPC purification). The DNAzyme origami was prepared under the same conditions as the normal DNA origami. The catalysis experiment was performed in HEPES buffer saline (NaCl 150 mM, 20 mM HEPES (Dojindo, 345–06681, lot ES132)) containing 1 mM zinc sulphate. Reactions were performed with one equivalent of 60 nM DNAzyme, corresponding to 5 nM DNA origami. The reaction mixture was then thermally annealed. First, it was heated to 40 °C and cooled to 25 °C at a rate of −0.5 °C min^−1^ using a PCR machine. The sample was then immediately run on native PAGE containing 12 wt% bis‐acrylamide, 1×TBE, at a constant voltage of 200 V, for 35 min. The gel was imaged using the FLA5000 image analyzer, ex. 532 nm, LPB filter. The software used to measure the band intensity of agarose gels was also used to measure the percentage of substrate converted into fragments. See Supporting results section 3.3.

## Conflict of interest

The authors declare no conflict of interest.

1

## Supporting information

As a service to our authors and readers, this journal provides supporting information supplied by the authors. Such materials are peer reviewed and may be re‐organized for online delivery, but are not copy‐edited or typeset. Technical support issues arising from supporting information (other than missing files) should be addressed to the authors.

Supporting Information

## Data Availability

The data that support the findings of this study are available in the supplementary material of this article.
