# Supplementary material for: Water‐in‐Water Droplets Selectively Uptake Self‐Assembled DNA Nano/Microstructures: a Versatile Method for Purification in DNA Nanotechnology
Source: Chembiochem. 2022 Jul 1;23(17):e202200240. doi: 10.1002/cbic.202200240 (PMC9544409; doi:10.1002/cbic.202200240)
Supplement: Supplementary file 1 — Supporting Information [file CBIC-23-0-s001.pdf]

ChemBioChem

Supporting Information

Water-in-Water Droplets Selectively Uptake Self-Assembled DNA Nano/Microstructures: a Versatile Method for Purification in DNA Nanotechnology

Marcos K. Masukawa, Yusuke Sato, Fujio Yu, Kanta Tsumoto, Kenichi Yoshikawa, and Masahiro Takinoue*

1. Supplementary materials and methods

1.1. Hexagonal DNA origami

The DNA origami with the shape of a hexagonal nanoplate was used to initially test the viability of W/W-droplet fractionation to purify DNA structures. The DNA origami was composed of six interconnected trapezoidal structures named P1-P6 (Fig. S1). Each trapezoidal portion contains staples in positions L1 and L2. We produced a version of the DNA origamis with staples in positions L1 and L2 extended with an 8-17 deoxirybozyme (DNAzyme), totaling 12 DNAzymes per DNA origamis. The table S1 shows the list of staples used for producing the non-enzymatic hexagonal DNA origami and their length, relative start/end in the scaffold and indicating whether they belong to the L1 or L2 position. . Table S2 indicates the staples used for producing the enzymatic DNA origami, showing only the staples that substitute those in positions L1 and L2.

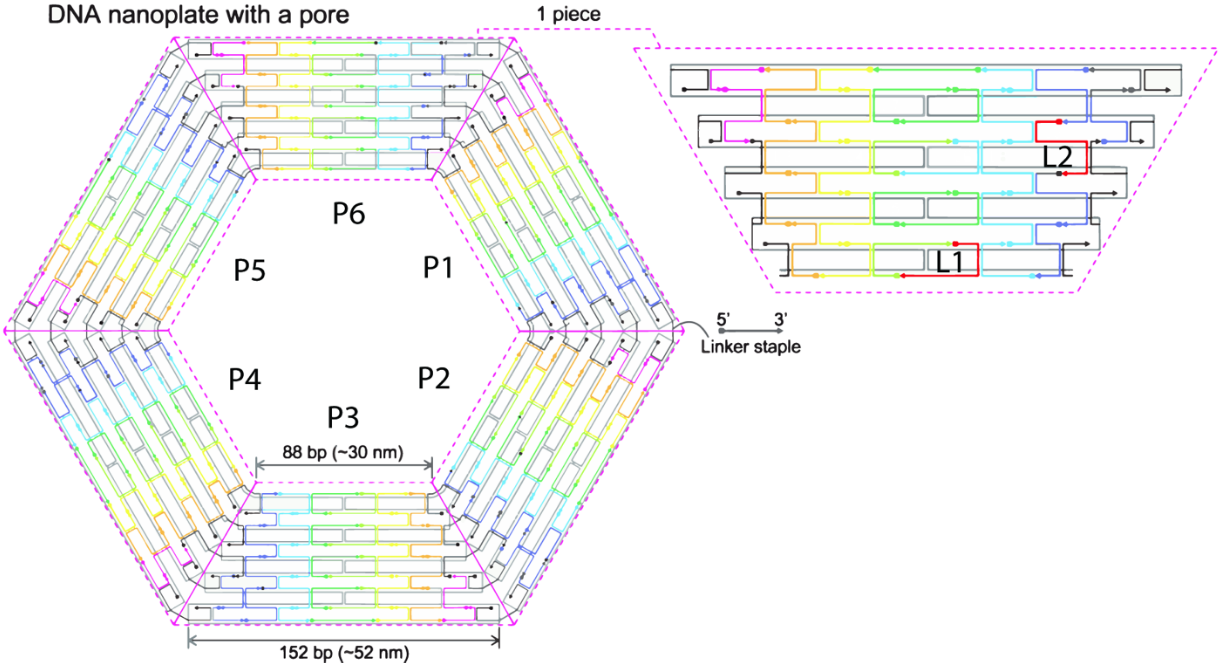

Supplementary Figure S1. Structure of hexagonal DNA origami, adapted from Ishikawa et al. [1]

Supplementary Table S1. The sequence of oligonucleotides used in the DNA origami with a hexagonal shape. The columns indicate the staple number, sequence, length, relative start and end, and indicate if the staple belongs to the L1 or L2 position indicated in Fig. S1.

Staple No.	Sequence (5' to 3')	Length (nt)	Start	End	Position
1	AAACAAAAAAGATGATGAAACAAAGAGAATAT	32	3[512]	4[496]	
2	CGAAACAAAGTACAACCGAAATCC	24	11[88]	12[96]	
3	AAATACCGTAAATTTAATGGTAAGCAGATAGCT TTTAAGA	40	1[432]	1[383]	
4	AAAAATCACGTCCAATACTGCGGACAAAATAGCG AGAGGC	40	17[160]	19[167]	
5	TCATTACCTAGGCTGGCTGACCTTGGCGCAGA	32	15[80]	13[79]	
6	GAAGGCACTAAAACACTCATCTTT	24	7[96]	8[88]	
7	GGCTTGCATGAATTTCTTAAACAGAAAAAGGC	32	4[79]	2[80]	
8	CTTGCTTCATTTCATTTGAATTACCGAATTAT	32	2[543]	4[544]	L2-P3
9	TCCCATCCAAGTCCTGAACAAGAATTTTACAT	32	6[495]	5[511]	
10	TATTACGCAGTATGTTATAGAAAA	24	0[295]	1[287]	
11	CGGAGAGGAGGCCGGAGACAGTCAGATAAAAA	32	17[272]	15[271]	L2-P5
12	AAGGAAACCGAGGAAATGAAATAGCAATAGCTT CAGAGGG	40	0[359]	2[352]	
13	AGATGGTTCATTGTGAATTACAGGTAGAAAGA	32	16[67]	18[60]	
14	AGAAACAACGCAATAATAACGGAA	24	1[336]	0[328]	
15	GAGGGGGAGTTAAAGGTCTTGACAAGAACTTG	32	3[68]	16[68]	
16	CGCAAAGACAACATATAAAATAGCAAGCCCAACA TTTTCA	40	1[240]	1[191]	
17	TAATGCGCAATGGCTATTAGGTCTGAGAGACTTCA AAATC	40	18[575]	1[575]	
18	ATCAATATATTGAGGAAGGTTATCCATTTGAG	32	16[527]	14[528]	
19	GTCTGGAAGTCAATAAAATAAATC	24	12[167]	12[264]	
20	GCTTAGGTTGGGTTATTAAGACGCTGAGAAGATGA ATAAC	40	0[551]	2[544]	
21	CTTTCCTTCATTACCGTTGCGGGA	24	7[456]	7[359]	
22	CCTCAGAGAGCCGCCACCCTCAGAGTATTCTA	32	8[303]	7[319]	
23	AGCGTCTTAAATAAGAAACGATTTTTTACAGA	32	5[336]	3[335]	
24	TAGTTAGCGTAACGATAGAATAGA	24	0[103]	1[95]	
25	GTTTGGAAGAGATAGGGTTGAGTGAGAGGTGA	32	18[495]	17[511]	
26	GTTGTACCGCCTCAGAGCATAAAGATGTGCTG	32	14[303]	13[319]	
27	TCATTTCACTGATTGCTTTGAATAGCGTAGAT	32	4[543]	6[544]	
28	GCTTAGATATAACTATATGTAAAT	24	1[528]	0[520]	

29	AATTGTGTGGAGATTTGTATCATCGAGTAGAT	32	12[111]	11[127]	L1-P6
30	TAAAAAATGAAACATAAATCAAAC	24	16[567]	3[567]	
31	CAAACAAAAATTTACCGTTCCAGTGTGTACTG	32	7[144]	5[143]	
32	GATTTAGAGTATTAAATCCTTTGCTTTTGCGG	32	14[527]	12[528]	
33	TTTTTAGATGTAATACTTTTGCGGAAGGCAAA	32	15[272]	13[271]	
34	TGATAGCCCTAAAGAGCCAGCAGCGCATCACC	32	18[563]	16[556]	
35	GAATTAGCTAACATCCCCTGTTTA	24	13[272]	11[279]	
36	GTAGGAATATCATTCCAGAAACCA	24	8[471]	6[464]	
37	GCTATATTTTCATTTGCTAATAGT	24	11[280]	12[288]	
38	CGATAGCAACTTGAGCCATTTGGGGGGAAGGT	32	4[271]	2[272]	
39	ATGGCAATCATTATCACCGAACGT	24	11[536]	13[543]	
40	GAGAATAACGCTAATATCAGAGAGTAAGAGCA	32	3[336]	1[335]	
41	GGTAAAGAAATGCAATGCCTGAGTCGGCACCG	32	16[303]	15[319]	
42	ATAAGTATAAGGATTAGGATTAGCAACAGTTA	32	2[159]	4[160]	L2-P1
43	TGAAAGGACTGGTCAGTTGGCAAACGCCTGGC	32	15[512]	16[496]	
44	CCCAATAAATAACCCACAAGAATTGACAAAAG	32	1[320]	2[304]	
45	TCCAAAAGCAGTTTCAGCGGAGTGCTAAAGTTTTG TCGTC	40	2[79]	0[72]	
46	CCTTCTGACCTGAAAGATACCGAA	24	19[520]	18[528]	
47	TAGTAAAATTGCCAGAGGGGTGTACCCCGGTTATG TCAAT	40	18[191]	18[240]	
48	ATTTCATCTTCTGACCACCG	20	0[455]	1[443]	
49	ATCATGGTACGGCCAGCAGGGTTT	24	11[344]	13[351]	
50	ACCAGAGCGAACCACCCATTAAAG	24	7[264]	7[167]	
51	TAAGAGCAACACTATCATAACCCTCGTTTACC	32	19[104]	19[135]	
52	CCCTCGAACAAAGTTACCAG	20	1[372]	0[360]	
53	AGGGCAGGCTGCGCAAGGGCGCCAGGGTCAGC	32	16[363]	16[452]	
54	ATCTACGTCCAGTCAGGACGTTGGATGCTTTA	32	18[111]	17[127]	
55	ACGGAATAAGTTACGGAAATTATTTCACCGTC	32	1[252]	3[259]	
56	TTCAATTCAACTAATGCAGA	20	18[59]	19[71]	
57	AAAGCCAAGGCGTTAAATAAGAATAAATATATTT TAGTTA	40	2[463]	0[456]	
58	CGAACCACGTATTAACACCGCCTGAACCCTCA	32	18[527]	16[528]	
59	CGCCGCCAGCATTGACGATATTCA	24	8[151]	7[143]	
60	GAAGTTTCGATCGTCACCCTCAGCGTCGCTGA	32	6[79]	4[80]	
61	AACAAAGATCCTGATTATCAGATG	24	12[527]	11[535]	
62	AAAAGGGACATTCTGGCCAACAGAGATAGAAC	32	19[488]	19[519]	
63	CCCTTAGGAACCCATGTACC	20	1[180]	0[168]	
64	GGCGACATGTTTACCAGCGCCAAAGAGTTAAG	32	2[303]	1[319]	
65	CCAAAAACAGGAAGATGAGAATCGATGAACGGAT TAATGC	40	19[264]	17[271]	

66	CCCGAAAGAAGCGAACCAGACCGGGAGGTCAT	32	16[143]	14[144]	
67	GGATGTCAGGATTAGATTTCAACGCAAGAATC	32	16[171]	16[260]	
68	AATCAAAATCAAGTTTGCCTTTAGGAAACCAT	32	6[271]	4[272]	
69	TGTAGCGCTATTAGCGTTTGCCATGAACCGCC	32	5[288]	7[287]	
70	TGTGATAAATAACGCTCAACAGTACGCCATAT	32	1[444]	3[451]	
71	AAGGAACAGTTGAAAATCTCCAAACTTGATAC	32	1[96]	3[95]	
72	GCAAACAATGTATAAGCAAATATT	24	18[287]	19[295]	
73	GACCCCCATACGTAATTTCATGAG	24	8[87]	6[80]	
74	AACAACTTTCAAGAGCCTTTAATTTTGCTTTC	32	1[60]	3[67]	
75	TACATAACGCCAAAAGCGGAACAACATTATTACC TTATGC	40	19[72]	17[79]	
76	GAGGCATTGACAAAAGGTAAAGTATTTATCAA	32	3[480]	5[479]	
77	CAATAGATTAATTTACGAGCATGTAAGAACGG	32	5[480]	7[479]	
78	CCGCCACCTCACCAGTACAAACTA	24	1[144]	0[136]	
79	TATAATGCATGGCTTAGAGCTTAAAGGACAGA	32	13[128]	14[112]	
80	CCAGCAAACGGAAACGTCACCAATCGTCAGAC	32	3[288]	5[287]	
81	TTATTTGCTTAACGTCAGATGAATACAATAAC	32	7[528]	5[527]	
82	TAATTGAGCATAAAAACAGGGAAGTTATTTAT	32	2[351]	4[352]	L2-P2
83	AACAGTTCATTGAATCCCCCTCAAGAAGAAAA	32	17[128]	18[112]	
84	TTGGCAGATTCACCAGAAGAACGTGGACTCCAGC AAAATC	40	19[456]	17[463]	
85	CATCATATAACCACCAGAAGGAGCACATACGA	32	11[512]	12[496]	
86	AAATATTCAGAAAACGAGAATGACAGAGGAAG	32	18[143]	16[144]	
87	TTTAACCTTTTTAACCTCCG	20	1[564]	0[552]	
88	AGCAAGCAAATCAGATAGGCGTTT	24	8[343]	7[335]	
89	AGTCTCTGTAAATCCTACCAGAGC	24	6[159]	8[152]	
90	TTTCCAGACGTTAGTAGCTA	20	0[71]	1[59]	
91	AGTAGCATAAAATTAAGCAATAAAAAAAACAT	32	12[287]	14[288]	
92	CTCAAGAGAGCCCGGAATAGGTGTCCTCAGAA	32	3[144]	1[143]	
93	GATTGTTTGGATTATATATCAAAA	24	8[535]	7[527]	
94	CAGACGACCAACGCCAACATGTAAACCAGTAT	32	4[463]	2[464]	
95	ATTTAACATGTAAATCGTCGCTATTAGCGATA	32	3[528]	1[527]	
96	CCGGGTACCTGCAGGTCGACTCTAGGTGGCAT	32	11[320]	12[304]	
97	GATTTTAACGAGTAGTAAATTGGGCCGGATAT	32	17[80]	15[79]	L2-P6
98	ATCGGCCAAGTGAGACGGGCAACAAGTTGCAG	32	14[479]	16[480]	
99	ACAGCATCGAGGACTAAAGACTTTGCCACTAC	32	5[96]	7[95]	
100	TATTAATTCCGTCAATAGATAATATAAAATAT	32	13[544]	15[543]	
101	CAGAGACTATTATCAACCGTTATG	24	16[183]	16[248]	
102	CACTATTATCACACGACCAGTAAT	24	18[479]	19[487]	
103	AATTGCGTTGGGGTGCTTTCCTGT	24	13[464]	11[471]	
104	AGCAAGGCATCACCAGTAGCACCAAATGAAAA	32	4[303]	3[319]	

105	AGTAACAAATTGACCGTAATGGGAGCCTCAGG	32	18[335]	16[336]	
106	GAATACACCAACCTAAAACGAAAGTCAGACGA	32	8[111]	7[127]	
107	TCCCTCAGCCACCACCCTCAGAGC	24	7[288]	8[280]	
108	CCTGTAGCCAGCAGATGGGCGCATCCAGTTTG	32	18[371]	16[364]	
109	ACCTTTAAAGTAAGGCGAGAAGCC	24	14[175]	14[256]	
110	TGGGAAGGACGACTAAGGTTTGCG	24	14[367]	14[448]	
111	CCCAATCCTCCAGAGCCTAATTTGAGCCTTAA	32	4[351]	6[352]	
112	TTTTGCAAAAGAAGTTTGTT	20	19[168]	18[180]	
113	GGTGAGAAGTAGCTATTTTTGAGAAGTCTGGA	32	16[287]	18[288]	
114	TTAAGACAATAAACAATAAACAGCCATACGCA	32	3[452]	3[363]	
115	CAAGCGGTATCAAAAGAATAGCCCCAAGAGTC	32	16[479]	18[480]	
116	TTTTGCGGTGTAGCTCAACATGTTCCATATAA	32	14[143]	12[144]	
117	GTATTAAACGTTTTTATTTTCATC	24	7[480]	8[472]	
118	GCTGATGCAAATCCAATCGCAAGACAAAGAAC	32	0[519]	0[488]	
119	TTGAGTAATCATCAATCAGAAATA	24	12[551]	7[551]	
120	TAGCAGCCTTTGTTTAACGTCAAATTACCATT	32	3[320]	4[304]	
121	ATCAAGATCTCCCGACCGCCCAAT	24	6[351]	8[344]	
122	CTTCTGGTACTCCAGCCAGCTTTCAATGTGTA	32	15[320]	16[304]	
123	ACATACATAAAGGTGGCACC	20	0[263]	1[251]	
124	GACAACTCAGTATTAGACTTTACACTGTCGTG	32	13[512]	14[496]	
125	GCGTCTGGCCATCAAAAATACTCAATCGTCTGA ACCGTCT	40	18[383]	18[432]	
126	CGCAAATGGTTTCATTTTAAATAT	24	11[152]	13[159]	
127	GCGAGAAAACTTTTTCAAACACCG	24	0[487]	1[479]	
128	GTAATAAGTGCCTATTTCGGAACCTGAGACTC	32	5[144]	3[143]	
129	ACCGCCACATCACCGTACTCAGGAATAATAAT	32	1[128]	2[112]	
130	TGCCTGAGGATCTACAAAGGCTATTGGGAACA	32	18[303]	17[319]	
131	AGGCGAAAGTTCCGAAATCGACGTCAAAGGGC	32	16[451]	18[444]	
132	GTTGGTGTTTTCATCAACATTAAATAAATCAGCT CATTTT	40	17[352]	19[359]	
133	TTTTTCACACTAAAGGAATTGCGAGGTTTAGT	32	2[111]	1[127]	
134	GCCGGAAGCTCACAATTCCACACAGGAATTAT	32	12[495]	11[511]	L1-P4
135	AAGATCGCGCCGGAAACCAGGCAATCTTCGCT	32	16[335]	14[336]	
136	AGACGACGATAAAAACATCGTCAT	24	19[136]	18[144]	
137	TTGGCCTTAGGAGGTTGAGGCAGGAGGCAAAA	32	7[128]	8[112]	L1-P1
138	TGAACGGTTGACCAACTTTGAAAGTTGCTGAA	32	14[111]	13[127]	
139	GCCCACGCGCGCCGACAATGACAAGAAAGTAT	32	4[111]	3[127]	
140	ATTACGCCTTAAGTTGGGTAACGCTGCCAAGC	32	14[335]	12[336]	
141	TAAGAGGCTATTATTCTGAAACATCAACCATC	32	3[128]	4[112]	
142	GAATCATACGTTATACAAATTCTTTTTAGGCA	32	1[480]	3[479]	
143	CCTGAGAGGCTGATTGCCCTTCACTCAACAGT	32	16[495]	15[511]	

144	TTCAGCTATCGGTTGACCAGTTAC	24	5[448]	5[367]	
145	ACCATCAATCTAGCTGATAATAATCGTAAAAC	32	16[259]	18[252]	
146	CAATTCTAGGGCGCGAGCTGAAAAGAGGATCC	32	12[303]	11[319]	L1-P5
147	ACAGACAATATTTTTGGAAC	20	19[552]	18[564]	
148	CAACGCCTGTAGCATTCCACAGACAGCCCTCA	32	0[135]	0[104]	
149	TTTTGCGGCATTACGACATCAAGA	24	5[64]	14[64]	
150	CAGTTGATACCATTAGATACATTT	24	12[143]	11[151]	
151	AGAACGCGATAGAAGGCTTATCCGACCGCCAC	32	7[320]	8[304]	L1-P2
152	AAATATTGTATTTTGTCACAATCAAGCAAACGT AGAAAAT	40	2[271]	0[264]	
153	GTAAAGCCTGCGCTCACTGCCCGCATTAATGA	32	12[479]	14[480]	
154	CTTTAGGATGAACCTCAAATATCACAACAGTG	32	15[544]	17[543]	
155	CAAGGCGAAGCTGGCGAAAGGGGGCTAAATCG	32	13[320]	14[304]	
156	TGAAAACATAATTAATTTTCCCTTTGTTTAGT	32	1[512]	2[496]	
157	TAGCGATAATCAGAAAAGCC	20	18[251]	19[263]	
158	CCACGCTGAACATCGCCATTAAAACGTAAGAATA CGTGGC	40	17[544]	19[551]	
159	ATGCCCCCTTTTAACGGGGTCAGTGAAAGCGC	32	4[159]	6[160]	
160	TCTGCGTAACCGTGATGGTGATCC	24	16[375]	16[440]	
161	GTAAAACGCATAGCTGCTAATGAG	24	12[359]	12[456]	
162	GAGCTTCAACTTCAAATATCGCGTAATAAGGC	32	15[128]	16[112]	
163	TTTCAGGTACGTAAAAATAATCCT	24	6[543]	8[536]	
164	GGATTCGCATTACCTGAGCAAAAGTTAATTAC	32	5[528]	3[527]	
165	GTAACACTGAGTTTCGCTCAGAACCGCCACCCGG GTTGAT	40	0[167]	2[160]	
166	ATCATATGATTACTAGAAAAAGCCAGAATCCT	32	2[495]	1[511]	
167	GATTAGAGTTAAAATTCCAAGTTA	24	14[559]	5[559]	
168	TTGCGCACTAACAACTATCGCGCAGAGGCTTT	32	16[555]	3[555]	
169	CGACAGAATCACAATGGCCTTGAG	24	5[256]	5[175]	
170	AAAGTACCTTCGAGCCAGTAATAACATCAAGA	32	4[495]	3[511]	
171	CAGGCGCACAAATCAACGTAACAAACGAGAAA	32	14[95]	16[96]	
172	TCCAACAGTGCATCAAAAAGATTACATAAATC	32	15[160]	17[159]	
173	TTTTCACCACGCGCGGGGAGAGGCCTCACATT	32	15[464]	13[463]	
174	TTAGACGGCCCTGAACAAAGATCTTACCGAAG	32	3[364]	1[371]	
175	GTGAAATTGTTATCCGCATAAAGT	24	11[472]	12[480]	
176	TTAGTTTGTCCCAATTCTGCGAACGCCTGATA	32	11[128]	12[112]	
177	GCGACCTGCATAAGGGAACCGAACGTACAGAC	32	12[95]	14[96]	
178	CTTACCAACCCAGCTACAATTTTATCGGTCAT	32	5[320]	6[304]	
179	CGCATTAAATTTTTGTTGTGAGCG	24	19[328]	18[336]	
180	ATTACATTAAAGGCGGATAAAGTA	24	3[248]	3[183]	
181	GGGTAAAAGCGATTATTACTTAGC	24	7[72]	12[72]	

182	TAAATTGTAAACGTTAATATTTTGTTAAAATT	32	19[296]	19[327]	
183	CGAACTAAGAATTACGAGGCATAG	24	18[95]	19[103]	
184	TTTAATGGAATATATGTGAGGTCAATAGTGAA	32	3[556]	1[563]	
185	ACCTACCACTTCTGAATAATGGAATCGAGAAC	32	7[512]	8[496]	L1-P3
186	ACCGGCACCGTAATCAAGTGCCCGTATAGGGG	32	3[260]	3[171]	
187	CGGTCAATCTCCATGTACCAAGCG	24	13[80]	11[87]	
188	AGCCCCCTGTTTTCATCGGCATTTTCCTGAAT	32	6[303]	5[319]	
189	AAGCAAGCCCAAGTACCGCACTCAGGGTTAGA	32	8[495]	7[511]	
190	CGATAGTTATAACCGATATATTCGAGCGAAAG	32	3[96]	5[95]	
191	TTAACCAATAGGAACGCCTT	20	19[360]	18[372]	
192	TTCATATGTCAACCGATTGAGGGAAATTAGAG	32	1[288]	3[287]	
193	GAATGGGCTTAAACTGAACAGAGA	24	3[440]	3[375]	
194	TAGCGAACTAGTTGCTATTTTGCACGCTAACG	32	7[336]	5[335]	
195	GCAACTAATTGCTCCTTTTGATAAAAGCAAAC	32	13[160]	15[159]	
196	TCCCAGTCGCGATCGGTGCGGGCCAGCGCCAT	32	13[352]	15[351]	
197	TAGACTGGATAGGGTCTTTACCCTAGCAAAGC	32	18[179]	16[172]	
198	ATACAGGAAAGCGTCATACATGGCCGGCTACA	32	5[128]	6[112]	
199	CCTTATAACCACGCTGGTTTGCCCGGTTTTTC	32	17[464]	15[463]	L2-P4
200	TACCCAAAAGAACTGGCATGATTAAGACTCCT	32	0[327]	0[296]	
201	CAGCGTATCGGTACTTTAATTAAT	24	3[56]	16[56]	
202	CGCCACCACACCACCGCTTTTCAT	24	8[279]	6[272]	
203	CCAGCTGCTTTCCAGTCGGGAAACAACAATTC	32	14[495]	13[511]	
204	GGCGGTCACAGCAGAAGATAAAACTTGTTCCA	32	17[512]	18[496]	
205	GGGATTTTAATGAATTTTCTTAGGAATACCACTC AGTTGA	40	1[48]	18[48]	
206	CGGGAGAAATACAGTAACAGTACCAAATAATA	32	5[512]	6[496]	
207	GAGGCTTTGGAACGAGGGTAGCAATTTTGATG	32	6[111]	5[127]	
208	CACCAGAAGAACTGGCTCATTATATAATAAAA	32	16[95]	18[96]	
209	TATGACCCACCCTCATATATTTTATTCAAAAG	32	14[287]	16[288]	
210	TTGCCCTGAGCTGCTCATTCAGTGTTTAATTC	32	16[111]	15[127]	
211	TTTTGCTCGTGCCGTCGAGATCAGAGCCACCA	32	3[172]	1[179]	
212	TTGCATGCCGAGCTCGAATTCGTA	24	12[335]	11[343]	
213	AACGGCGGCCCGTCGGATTCTCCGCAGGTCAT	32	17[320]	18[304]	
214	GAAAAAATGGATTATTTACA	20	18[443]	19[455]	
215	TCGCCATTGACGACGACAGTATCGTAGGTCAC	32	15[352]	17[351]	
216	ATCAATAAATGCAGAACGCGCCTGATTCTGTC	32	6[463]	4[464]	

Supplementary Table S2. The sequence of oligonucleotides used to add the enzymatic activity to the hexagonal DNA origami. The staples listed below contain the 8-17 deoxirybozyme at the 3' end and substitute the staples listed in Table S1, which occupy the positions L1 and L2.

**Position**	**Sequence**
**L2-P3**	CTTGCTTCATTTCATTTGAATTACCGAATTATTTATTATTATCTCTTCTCCGAGCCGGTCGAAATAGTGAAAA
**L2-P5**	CGGAGAGGAGGCCGGAGACAGTCAGATAAAAATTATTATTATCTCTTCTCCGAGCCGGTCGAAATAGTGAAAA
**L1-P6**	AATTGTGTGGAGATTTGTATCATCGAGTAGATTTATTATTATCTCTTCTCCGAGCCGGTCGAAATAGTGAAAA
**L2-P1**	ATAAGTATAAGGATTAGGATTAGCAACAGTTATTATTATTATCTCTTCTCCGAGCCGGTCGAAATAGTGAAAA
**L2-P2**	TAATTGAGCATAAAAACAGGGAAGTTATTTATTTATTATTATCTCTTCTCCGAGCCGGTCGAAATAGTGAAAA
**L2-P6**	GATTTTAACGAGTAGTAAATTGGGCCGGATATTTATTATTATCTCTTCTCCGAGCCGGTCGAAATAGTGAAAA
**L1-P4**	GCCGGAAGCTCACAATTCCACACAGGAATTATTTATTATTATCTCTTCTCCGAGCCGGTCGAAATAGTGAAAA
**L1-P1**	TTGGCCTTAGGAGGTTGAGGCAGGAGGCAAAATTATTATTATCTCTTCTCCGAGCCGGTCGAAATAGTGAAAA
**L1-P5**	CAATTCTAGGGCGCGAGCTGAAAAGAGGATCCTTATTATTATCTCTTCTCCGAGCCGGTCGAAATAGTGAAAA
**L1-P2**	AGAACGCGATAGAAGGCTTATCCGACCGCCACTTATTATTATCTCTTCTCCGAGCCGGTCGAAATAGTGAAAA
**L1-P3**	ACCTACCACTTCTGAATAATGGAATCGAGAACTTATTATTATCTCTTCTCCGAGCCGGTCGAAATAGTGAAAA

1.2. PURIFICATION OF DNA ORIGAMI BY MOLECULAR WEIGHT CUT-OFF FILTRATION AND GEL FILTRATION

Purification of DNA origami was performed without optimizations by molecular weight cut-off (MWCO) filtration and gel filtration to compare these purification methods to W/W-droplet fractionation.

In the purification by MWCO, a filter with a defined pore size is used. The samples are centrifuged in a cartridge whose walls are composed of this filter. Upon centrifugation, structures smaller than the pore-size pass through the filter, while larger structures such as DNA origami are kept in the cartridge. The DNA origami is then recovered by centrifuging the inverted cartridge. We used Amicon ultrafilters with 0.5 mL capacity and 100 kDa pore size (Merck Millipore, lot R0PB46526). We inserted the cartridge in the affiliated tube and loaded the cartridge with 400 μL of folding buffer (1×TAE buffer, 10 mM magnesium acetate). We then added 10 μL of the hexagonal DNA origami solution with 15 nM concentration. The cartridge in the affiliated tube was centrifuged at 15,000×g for 10 min at room temperature using a Himac CT15RE centrifuge, and the filtered solution was discarded. Another 400 μL of folding buffer was added to the cartridge, and the process was repeated thrice. To recover the DNA origami, the filter was inverted in a new affiliated tube and centrifuged at 1,000×g for 2 min using the same centrifuge. For comparing the relative band fluorescence intensity of purification by agarose electrophoresis, 20% of the initial sample volume was compared with 20% of the volume recovered after purification.

In the purification by gel filtration, the DNA origami solution is centrifuged through a layer of gel beads in a spin column. The beads have a defined pore size so that small structures such as the unbound staples have a long traveling distance, while larger structures have a shorter traveling distance and are recovered first during centrifugation. We used Sephacryl S-300 HR (Sigma Aldrich, 65546-95-4) to perform gel filtration. The polymer beads were diluted into a folding buffer in the proportion 1:3. The folding buffer was exchanged 3 times after the structures sedimented. Micro Bio Spin chromatography columns (Bio-rad, #7326204) were placed inside host tubes, and 630 μL of Sephacryl gel suspension was pipetted in the column. The columns inside the host tubes were centrifuged at 1,000×g at room temperature using a centrifuge HIMAC CT15RE. The buffer was discarded after centrifugation. This process was repeated 3 times until the Sephacryl formed a uniform packed layer inside the column. Then, 5 μL of hexagonal DNA origami at a concentration of 15 nM was placed on the top of the Sephacryl layer

and centrifuged at 1,200×g for 5 min using a centrifuge Kubota 3740. The solution was collected in the host tube. For comparing the purification yield by agarose electrophoresis, 20% of the initial sample volume was compared with 20% of the volume recovered after purification.

1.3. Additional DNA self-assembled structures

After testing the purification of hexagonal DNA origami by the W/W-droplet fractionation, we tested the possibility of purifying additional DNA self-assembled structures to determine the size boundaries in which W/W-droplet purification was viable. The structures produced and their approximate size are described in Table S3. In the next section, we describe the preparation protocol of each structure.

Supplementary Table S3. List of DNA self-assembled structures purified by W/W-droplet fractionations, indicating the approximate size of the structure.

	Structure	Description	Size
DNA origami and its components	M13	circular, single-stranded DNA from the M13mp18 virus, p7249 scaffold	~100 nm diameter
	staples	set of 216 staples used to form the hexagonal DNA origami [1]	~2.5 to 10 nm hydrodynamic diamter
	hexagon	2D DNA origami based on the p7249 scaffold with a hexagon shape	100 nm length
DNA motifs	Sticky-end-less Y-motif	branched structure with three stems[2]	~10 nm hydrodynamic diameter
	DNA hydrogel	networked of branched motifs containing an interacting sequence at the end of each branch[2]	~1 to 10 µm diameter
DNA origami with different scaffolds	triangle	2D DNA origami based on the p7249 scaffold with a triangular shape [3];	135 nm length
	mini-triangle	2D DNA origami based on the reduced scaffold M1.3 with a triangle shape[4]	~15 nm length
	24HB	3D DNA origami based on the expanded scaffold p7560 with a cylindrical shape formed by 24 helix bundles [5]	~80 nm length
DNA microtubes	4HT	DNA tubes formed by a bundle of 4 tile tubes [6]	several µm length
	14HT	DNA tubes formed by a bundle of 13 tile tubes [6]	several µm length

1.3.1. M1.3 DNA origami

The preparation of the DNA origami using the reduced scaffold followed the protocol by Said et al. [4] In this protocol, a linear section of the circular M13mp18 ssDNA (Tilibit Nanosystems type p7249 lot M1-1-5) was excised enzymatically, purified by agarose gel electrophoresis and cyclized by intramolecular ligation using an asymmetric template oligonucleotide. The resulting circular DNA is called M1.3 scaffold and is approximately one-tenth of the original scaffold in length. For the excision protocol, we used the restriction enzyme EcoRI (New England Biolabs inc. Code no.49889-67), restriction enzyme BglII (New England Biolabs inc. Code no.49890-69), and oligonucleotides CGAGCTCGAATTCGTAATCA for EcoRI digestion (Eurofins genomics K.K.) and TTTTGAGAGATCTACAAAGG for BglII digestion (Eurofins genomics K.K.). The cyclization protocol was slightly modified from the original paper[4]. In the cyclization step, a sample (288 µL) of linear M1.3 ssDNA(10 pmol) and an asymmetric template oligonucleotide GCTCGAATTTACAAAGGCTATCAGGTCATTGC (50 pmol) in T4 DNA Ligase Buffer (TAKARA BIO) containing 66 mM Tris-HCl, pH 7.6, 6.6 mM $MgCl_2$, 10 mM dithiothreitol (DTT) and 0.1 mM ATP were heated at 85°C for 5min and cooled to 20°C for 2h. Then 12 µL of T4 DNA Ligase (560 units) (TAKARA BIO) was added and incubated at 4°C for 18h. The reaction was stopped by heating at 85°C for 5 min. The solution was diluted by adding 200 µL of TE buffer, and excess template oligonucleotides were removed by ultrafiltration using Amicon Ultra 0.5 mL, 30 kDa (Merck Millipore). The filter was washed thrice with 300 µL of TE buffer, and the circular M1.3 scaffold was collected. Cyclization was confirmed by 1.5% agarose electrophoresis and 8% denaturing PAGE (bis-acrylamide solution from Nacalai Tesque, 06140-45). The gel contained 7M urea (Fujifilm-Wako chemicals Japan, 211-01213). For the formation of DNA origami using the M1.3 scaffold, 50 nM of the scaffold was annealed with 150 nM staple strands (Eurofins genomics K.K.) in 5 mM Tris-HCl, pH 8, 1 mM EDTA, 12 mM $MgCl_2$. The folding solution was heated to 85°C, then cooled down to 4°C at the rate of -0.8°C min^{-1}.

1.3.2. Triangle, 24HB DNA origami

The staples of the triangular DNA origami were obtained from the original report by Rothemund [3] and were prepared under the same conditions as the hexagonal DNA origami using staples from Eurofins Genomics (salt-free grade). The 3D DNA structure 24HB was composed of 10 nM scaffold p7560 (Tilibit Nanosystems, lot M1-3-4), 100 nM of staples (OPC grade from Eurofins genomics) as designed by Kuzbyk et al. [5], 1×TAE buffer and 16

mM magnesium acetate. The annealing program consisted of heating the sample to 80°C for 10 min and cooling it from 80°C to 65°C at -1°C min^{-1}, 65°C to 45°C at -0.1°C min^{-1} and 45°C to 25°C at -0.05°C min^{-1}.

1.3.3. STICKY-END-LESS Y-MOTIF AND DNA HYDROGEL

The Y-motif structure is a kind of DNA motif composed of a set of 3 oligonucleotides. We used Y-motifs that were both unable to form DNA hydrogel (sticky-end-less Y-motif) and those which were able to form a hydrogel, the difference being a palindromic sequence of 8 nucleotides (sticky-end) at the 5' end of the oligonucleotides. Motifs lacking sticky-ends could not form a polymeric network and remained in solution as individual units. The oligonucleotides used for making sticky-end-less Y-motifs and DNA hydrogels are displayed in Table S4. They were obtained from Eurofins Genomics (salt-free grade) and mixed with 1×TAE buffer, 100 mM potassium chloride, 10 mM sodium chloride, 0.5 mM magnesium chloride, and 0.1 mM calcium chloride. The sample was heated to 95°C for 3 minutes, then cooled to 25°C at -1°C min^{-1}.

1.3.4. 4HT AND 13HT MICROTUBES

The 4HT and 13HT microtubes were composed of bundles of nanotubes assembled using DNA tile technology. The bundles varied in number of nanotubes, which created microtubes with different diameters. Microtubes with bundles of 4 and 13 nanotubes, called 4HT and 13HT, respectively, were made following the protocol of Yin et al. [7]. A mixture of staples from Eurofins genomics (OPC grade) with the final concentration of 1 μM each and 1×TAE buffer and 12 mM magnesium acetate was annealed by heating to 80°C for 10 min and cooling from 80°C to 65°C at -1°C min^{-1}, 65°C to 45°C at -0.1°C min^{-1} and 45°C to 25°C at -0.05°C min^{-1}.

Supplementary Table S4. Oligonucleotide sequences used for making sticky-end-less Y-motif and DNA hydrogel [2].

	Oligonucleotide name	Sequence (5' to 3')
**Sticky-end-less Y-motif**	Y1 Sticky-end-less	CAGTGAGGACGGAAGTTTGTCGTAGCATCGCACC
	Y2 Sticky-end-less	CAACCACGCCTGTCCATTACTTCCGTCCTCACTG
	Y2 Sticky-end-less	GGTGCGATGCTACGACTTTGGACAGGCGTGGTTG
**DNA hydrogel**	Y1	GCTCGAGCCAGTGAGGACGGAAGTTTGTCGTAGCATCGCACC
	Y2	GCTCGAGCCAACCACGCCTGTCCATTACTTCCGTCCTCACTG
	Y3	GCTCGAGCGGTGCGATGCTACGACTTTGGACAGGCGTGGTTG

2. SUPPLEMENTARY THEORETICAL NOTES

2.1. DERIVATION OF PURIFICATION YIELD

During the purification protocol, the dextran-rich droplets were separated by centrifugation. The bottom phase is the denser dextran, and the top phase is the less dense PEG. The PEG phase is then exchanged, leaving the dextran solution and part of the PEG phase in the tube. The following model takes this process into account to calculate how much of a structure remains after each purification round. By applying n number of rounds, it is possible to obtain the purification yield.

Suppose a structure X that has a partition coefficient $K = [X]_{\mathrm{PEG}}/[X]_{\mathrm{Dex}}$, where $[X]_{\mathrm{PEG}}$ is the concentration in the PEG phase and $[X]_{\mathrm{Dex}}$ is the concentration in the dextran phase. The total concentration of X can be written considering both phases contain an amount of the structure and $[X] = (V_{\mathrm{PEG}}[X]_{\mathrm{PEG}} + V_{\mathrm{Dex}}[X]_{\mathrm{Dex}})/(V_{\mathrm{T}})$, where V_{PEG} is the volume of the PEG phase, V_{Dex} is the volume of the dextran phase and $V_{\mathrm{PEG}} + V_{\mathrm{Dex}} = V_{\mathrm{T}}$. By applying the definition of the partition coefficient, it is possible to isolate $[X]_{\mathrm{PEG}}$ and $[X]_{\mathrm{Dex}}$, obtaining respectively: $[X]_{\mathrm{Dex}} = [X]V_{\mathrm{T}}/(V_{\mathrm{Dex}} + V_{\mathrm{PEG}}K)$ and $[X]_{\mathrm{PEG}} = [X]V_{\mathrm{T}}/(V_{\mathrm{Dex}}/K + V_{\mathrm{PEG}})$.During the purification, after the removal of part of the PEG, there is a remaining volume. This volume can be adjusted and might be different at each purification round. In the protocol used in the experiments, the total W/W-droplet dispersion volume was 40 µL and at each round, 25 µL of the PEG phase were exchanged, which means 15 µL of the bottom remained after each purification round. In the last round, 5 µL were recovered so the volume was the same as the initial sample. We called the volume that remained at the bottom at each round$f_1.,f_2,f_3,\dots,f_{\mathrm{n}} > \mathrm{V}_{\mathrm{Dex}}$. We can rewrite the concentration of the structure X after the first purification round (Y^1) considering the ratio of the substance that was recovered:

$$Y^1 = \frac{[X]_{\mathrm{Dex}}V_{\mathrm{Dex}} + [X]_{\mathrm{PEG}}(f_1 - V_{\mathrm{Dex}})}{[X]V_{\mathrm{T}}}$$

The above equation can be written in terms of $[X]$ by inserting the equations for $[X]_{\mathrm{PEG}}$ and $[X]_{\mathrm{Dex}}$:

$$Y^1 = \frac{\frac{[X]V_{\mathrm{T}}}{V_{\mathrm{Dex}} + V_{\mathrm{PEG}}K}V_{Dex} + \frac{[X]V_{\mathrm{T}}}{\frac{V_{\mathrm{Dex}}}{K} + V_{\mathrm{PEG}}}(f_1 - V_{\mathrm{Dex}})}{[X]V_{\mathrm{T}}}$$

Which can be rewritten as:

$$Y^1 = \frac{V_{\mathrm{Dex}}}{V_{\mathrm{Dex}} + V_{\mathrm{PEG}}K} + \frac{f_1 - V_{\mathrm{Dex}}}{V_{\mathrm{Dex}}/K + V_{\mathrm{PEG}}}$$

On the nth purification round:

$$Y^n = \frac{V_{\text{Dex}}}{V_{\text{Dex}}x + V_{\text{PEG}}K} + \frac{f_n - V_{\text{Dex}}}{V_{\text{Dex}}/K + V_{\text{PEG}}} Y^{n-1}$$

By recursively applying this equation, the nth round can be rewritten:

$$Y^n = \prod_{i=1}^{n} \left(\frac{V_{\text{Dex}}}{V_{\text{Dex}}+V_{\text{PEG}}K} + \frac{f_i - V_{\text{Dex}}}{V_{\text{Dex}}/K + V_{\text{PEG}}} \right) \qquad \text{Equation S1}$$

which gives the purification yield by W/W-droplet fractionation for a structure given the parameters ($V_{\text{PEG}}, V_{\text{Dex}}$), the experimental protocol ($f_1, f_2, \ldots, f_{\text{n}}$) and the structure partition coefficient (K).

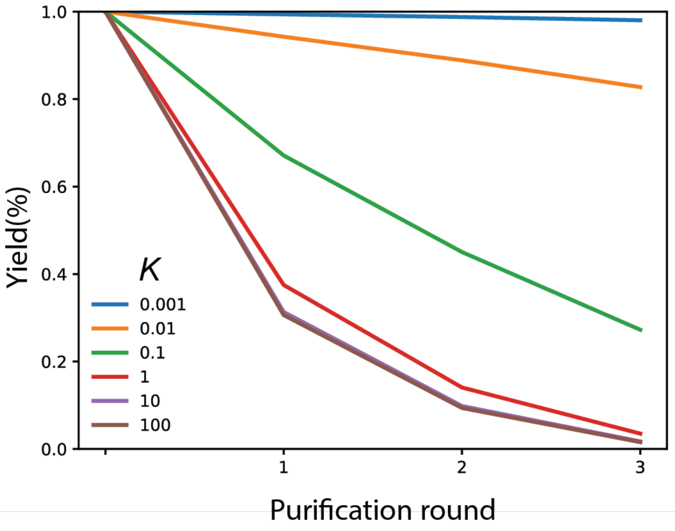

Supplementary Figure S2. Purification yield after each purification round by W/W-droplet fractionation for molecules and structures with different partition coefficients K. Parameters used: V_{Dex}= 5 µL, V_{PEG}= 35 µL, f_1, f_2, f_3= 15, 15, 5 µL

2.2 THEORETICAL SEPARATION EFFICIENCY

We numerically investigated the dependence of size on the purification efficiency of the DNA nano/microstructures, the efficiency being defined by Equation 1. To this end, we have adopted des Coundres' theory[8]. Des Coudres' theory is a general explanation for the distribution of structures in aqueous two-phase systems. It assumes that the complex interactions between the molecules, structures, and the polymer phases can be summarized by the interfacial free energy, which is given by $F = A\gamma$, where F is the interfacial free energy, A is the structure/molecule surface area, and γ is the surface tension between the structure/molecule and the polymer solution. When the structure/molecule is dispersed in PEG, we use the notation $F_{\text{PEG}} = A\gamma_{\text{PEG}}$, and when

the structure/molecule is in the dextran phase, $F_{\mathrm{Dex}} = A\gamma_{\mathrm{Dex}}$. The partition coefficient (K) of a structure/molecule is given by[9]:

$$K = \frac{[\mathrm{X}]_{\mathrm{PEG}}}{[\mathrm{X}]_{\mathrm{Dex}}} = e^{\frac{-(F_{\mathrm{PEG}} - F_{\mathrm{Dex}})}{k_B T}} = e^{\frac{-A\,\Delta\gamma}{k_B T}} \qquad \text{Equation S2}$$

$K \ll 1$ indicates that the structure/molecule is portioned primarily in the dextran phase; $K \gg 1$ that the structure/molecule is partitioned primarily in the PEG phase; and $K{\sim}1$ that the structure/molecule is present in similar concentrations in both phases. Particles/molecules with sufficiently different partition coefficients K can be separated by W/W-droplet fractionation; i.e., DNA nano/microstructures to be purified should have $K \ll 1$, and contaminants (excess DNA components) to be removed should have $K{\sim}1$. Eq. 1 relates the difference in surface tension between the structure/molecule and each polymer phase ($\Delta\gamma$), the structure/molecule surface area, and the partition coefficient K. Therefore, to obtain the partition coefficient, it is necessary to estimate $\Delta\gamma$and A.

An estimate for $\Delta\gamma$ can be made if we consider a model in which the polymers are ideal and non-interacting, the polymer solution is dilute, and the structures are much larger than the polymers. In this case, the surface tension between the molecule, structure, and the polymer solution can be approximated as the surface tension of a hard wall and a polymer solution, given by the formula[10]:

$$\gamma(\rho) = \frac{2\rho R_{\mathrm{g}}}{\sqrt{\pi}} k_{\mathrm{b}} T \qquad \text{Equation S3}$$

where ρ is the numeric concentration of the polymer and R_{g} is the polymer radius. To determine the numeric density of the polymers ρ, we assumed that the concentration of PEG was approximately 100 g L^{-1} and that of dextran was 200 g L^{-1}, based on the binodal curve of similar dextran-PEG ATPS[11], which is 9.6×10^{24} molecules m^{-3} in the PEG phase and 6×10^{23} molecules m^{-3}. We considered the size of PEG ($R_{\mathrm{g}} = 2.2$ nm)[12] and dextran ($R_{\mathrm{g}} =$ 9 nm) (formula for molecular weight to size provided by Aimar et al. [13]). Based on these values, $\gamma_{\mathrm{PEG}} = 97.9$ μN m^{-1}, $\gamma_{\mathrm{Dex}} = 25.04$ μN m^{-1} and $\Delta\gamma = 72.8$ μN m^{-1}.

By knowing $\Delta\gamma$, the structure/molecule surface area, and the conditions of the dextran-rich droplets, one can calculate K and the purification yield. The equation S1 gives the purification yield.

If a mixture of two different molecules/structures, one large and one small, is subjected to the W/W-droplet purification to remove one of them, the purification efficiency (E) can be defined as in Equation 1. To calculate the purification efficiency

(E), the purification yield is calculated based on the partition coefficient of each structure and the parameters of the dextran-rich droplets.

Fig. 4A shows the separation efficiency for a pair of target structures and contaminant molecules after 3 rounds of purification with parameters V_{Dex} = 5 µL, V_{PEG} = 35 µL,f_1, f_2, f_3= 15, 15, 5 µL, where the contaminant molecule has a surface area $A_{\mathrm{contaminant}}$ and the target structure has a surface area A_{target} and Δɣ = 72.8 µN m^{-1} for both contaminants and targets. It can be observed that efficient separation occurs only within a range of sizes.

3. SUPPLEMENTARY RESULTS

3.1 QUANTIFICATION OF AGAROSE ELECTROPHORESIS

The agarose electrophoresis provided information on the electrophoretic mobility of molecules and structures, which is closely related to their size, and allows the quantification of the extent of purification and the structural integrity of the structures. The images of the agarose electrophoresis were analyzed with ImageJ[14,15] and the intensity profile of each lane was taken. The intensity profiles of the agarose gel displayed in Fig. 2C are shown in Fig. S2. The intensity profile was then used to calculate the intensity of each band in the lane. The experiment was done in duplicate, and the average and standard error of each band for each lane were produced, which yielded Fig. 2D.

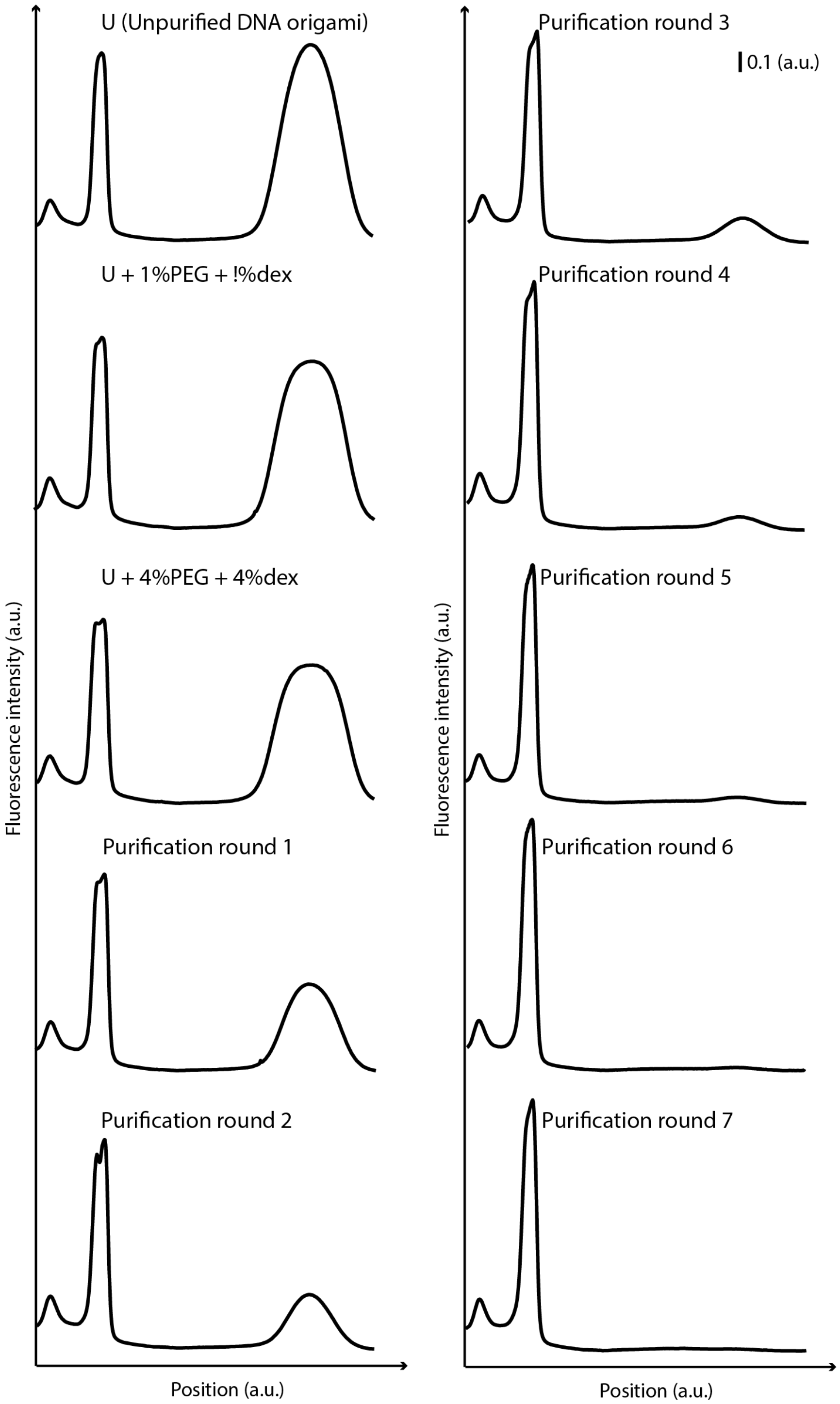

Supplementary Figure S3. Normalized fluorescence intensity profiles of agarose electrophoresis shown in Fig. 2C.

3.2 Purification by molecular weight cut-off filtration and gel filtration

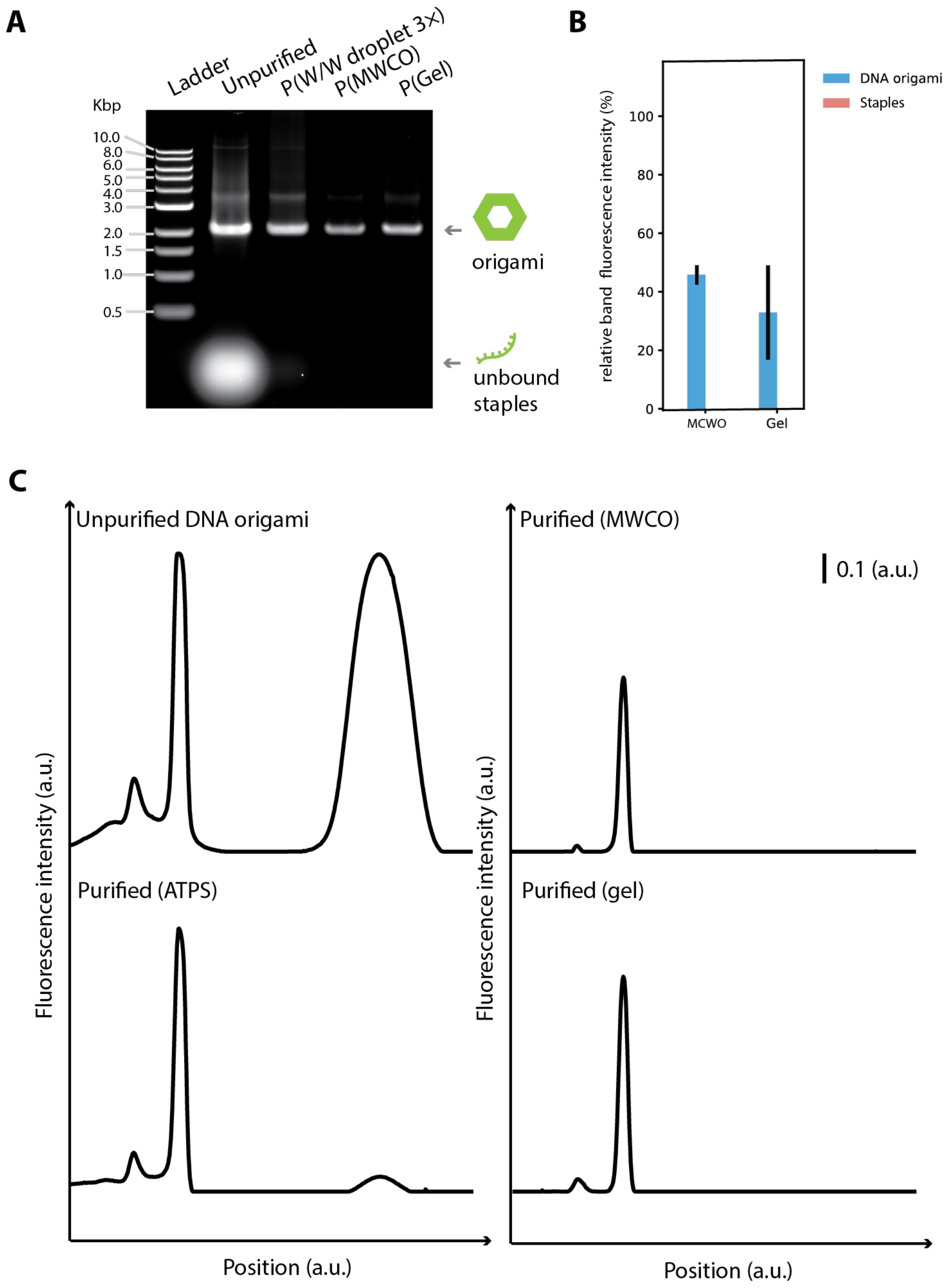

Supplementary Figure S4. Purification of hexagonal DNA origami by Amicon and gel filtration. **(A)** Agarose electrophoresis of Unpurified DNA origami (U) compared to DNA origami purified (P) by W/W-droplet fractionation, molecular weight cut-off, and gel filtration. Ladder = 1 kbp ladder. **(B)** Intensity profiles of the agarose electrophoresis for molecular weight cut-off and gel filtration. **(C)** Normalized fluorescence intensity profiles of agarose electrophoresis shown in Fig. S4A.

In this study, we focused on purifying low volume, low concentration samples purification of DNA origami. Under these conditions, W/W-droplet purification demonstrated to be a facile and high-yielding method. We compared the same conditions to molecular weight cut-off and gel filtration. Although Amicon and gel filtration can be high yielding methods, without optimization, these methods provided yields below 50%, as shown in Fig. S4.

3.3 DNA ORIGAMI WITH DNAZYME ACTIVITY

We fabricated two different kinds of DNA origami, one without catalytic activity and one with catalytic activity. The difference between these two designs is that in the catalytic DNA origami, there are 12 staples in positions L1 and L2 (see Fig. S1), which were extended with an 8-17 deoxyribozyme, which can cleave a DNA-RNA chimeric substrate. For most purification experiments, DNA origami without catalytic activity was used. The DNA origami with catalytic activity (DNAzyme origami) was used to test if the purification methods affected the functional DNA origamis due to residual polymers.

The catalytic DNAzyme origami was purified with molecular weight cut-off and W/W-droplet fractionation, while the non-catalytic DNA origami was purified with W/W-droplet fractionation. We observed the structural integrity of the DNA origami by agarose electrophoresis, as shown in Fig. S5. Prior to purification, both the DNAzyme and the DNA origami display a sharp band; however, after purification, the DNAzyme origami displays a smeared band. This occurs both when purified by molecular weight cut-off filtration and W/W-droplet fractionation. This likely indicates that the extended staples can induce partial aggregation of the DNAzyme origami.

We tested the catalytic activity of the DNAzyme origami purified by molecular weight cut-off and W/W-droplet fractionation on a DNA-RNA chimera substrate. As a negative control, a dummy molecule composed only of DNA was used. Free DNAzymes acted as a positive control, and DNA origami without catalytic activity was used as an additional negative control. The result is displayed in Fig. S6A. This figure shows that the free DNAzyme, the DNAzyme origami purified by molecular weight cut-off, and DNAzyme origami purified by W/W-droplet fractionation retain their enzymatic function. Although it may be seen that the DNAzyme purified by W/W-droplet fractionation has a low activity due to the high fluorescence of the non-cleaved substrate, when the intensity profile of the lanes was quantified, we observed that the lanes where W/W-droplet purification was used had a fluorescence intensity one order of magnitude higher than lanes whose samples were not

purified by W/W-droplet fractionation. That is, the W/W-droplet purification caused an increase in the fluorescence of the TAMRA dye, which makes it seem that the activity of DNAzyme origami purified by W/W-droplet fractionation is smaller than the DNAzyme origami purified by molecular weight cut-off. However, when the intensity of the lanes is normalized, and the relative intensity of bands is quantified, producing Fig. 3C, we observe that, in fact, free DNAzyme, DNAzyme origami purified by molecular weight cut-off, and DNAzyme origami purified by W/W-droplet fractionation have similar catalytic activity.

We confirmed that the W/W-droplet purification increases the fluorescence of the TAMRA dye by adding polymers dextran and PEG to the free DNAzyme and DNAzyme origami purified by molecular weight cut-off, as shown in Fig. S6B. In this figure, we can observe that the addition of polymers increases the fluorescence of the dye in more than one order of magnitude. When the intensity of the bands is normalized, we can confirm that the presence of polymers, nevertheless, does not affect the catalytic activity of the DNAzyme or the DNAzyme origami.

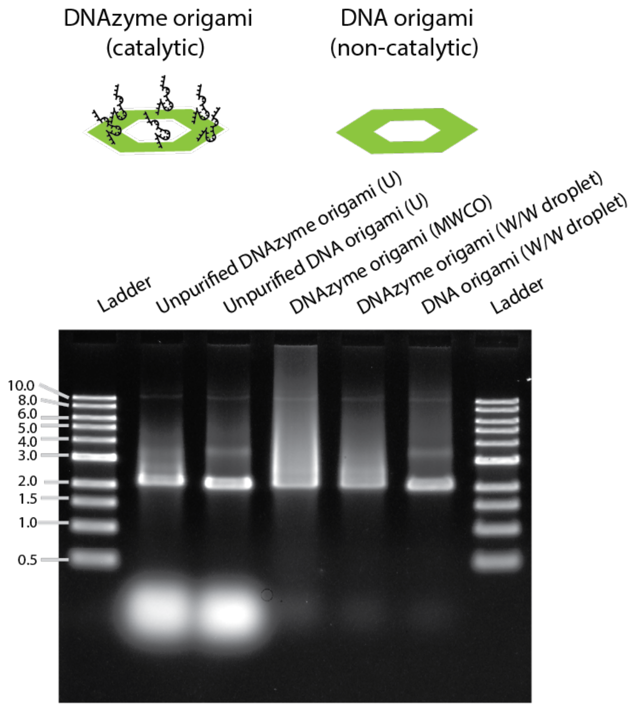

Supplementary Figure S5. Agarose electrophoresis of DNA origami modified with 8-17 deoxyribozyme (DNAzyme origami) and non-catalytic DNA origami (DNA origami), unpurified (U) and purified by either molecular weight cut-off filtration (MWCO) or W/W-droplet fractionation. Ladder = 1 kbp ladder.

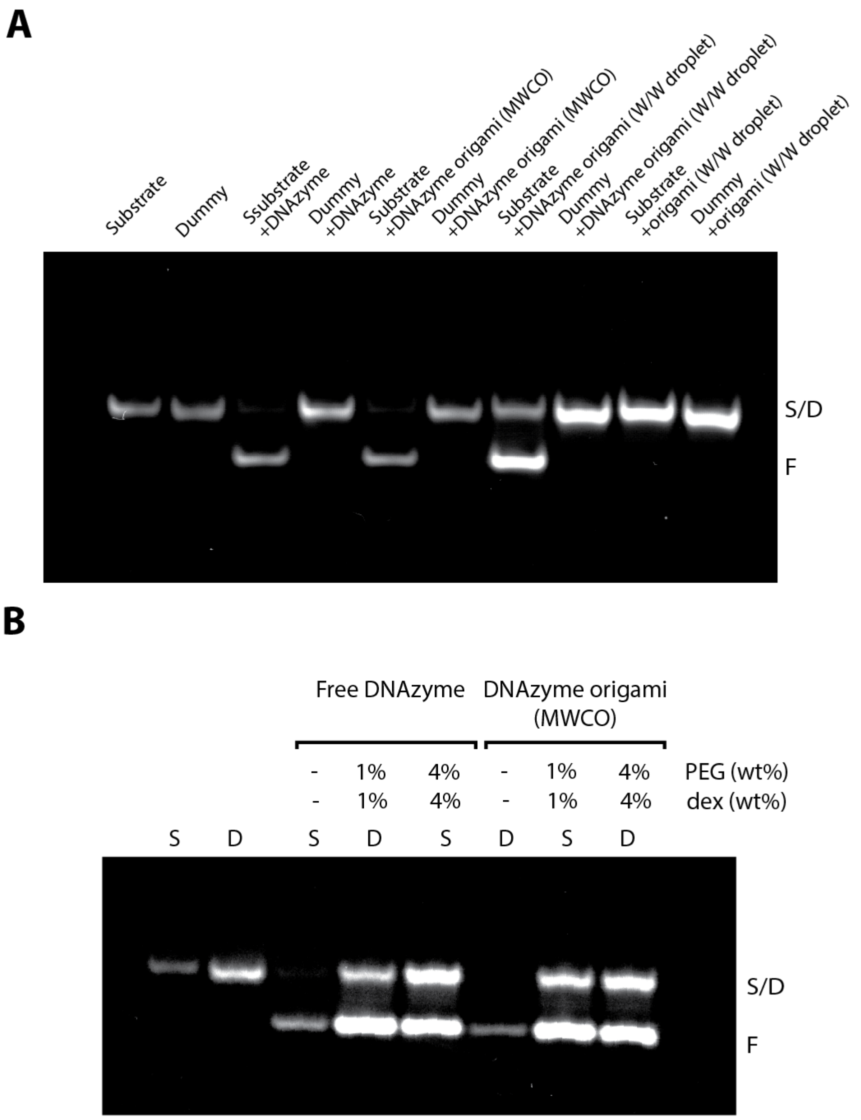

Supplementary Figure S6. The catalytic activity of DNAzyme origami. **(A)** Effect of purification method on the activity of DNAzyme origami. **(B)** Effect of polymer addition on the fluorescence intensity and catalytic activity of DNAzyme. S = substrate, D = dummy, F = fragment from substrate cleavage.

3.4 Purification of additional DNA self-assembled structures

We demonstrated that the W/W-droplet fractionation could be used to purify the structures listed in Table S3. The purification is confirmed by agarose electrophoresis, whose lane intensity profiles are plotted in Fig. S7.

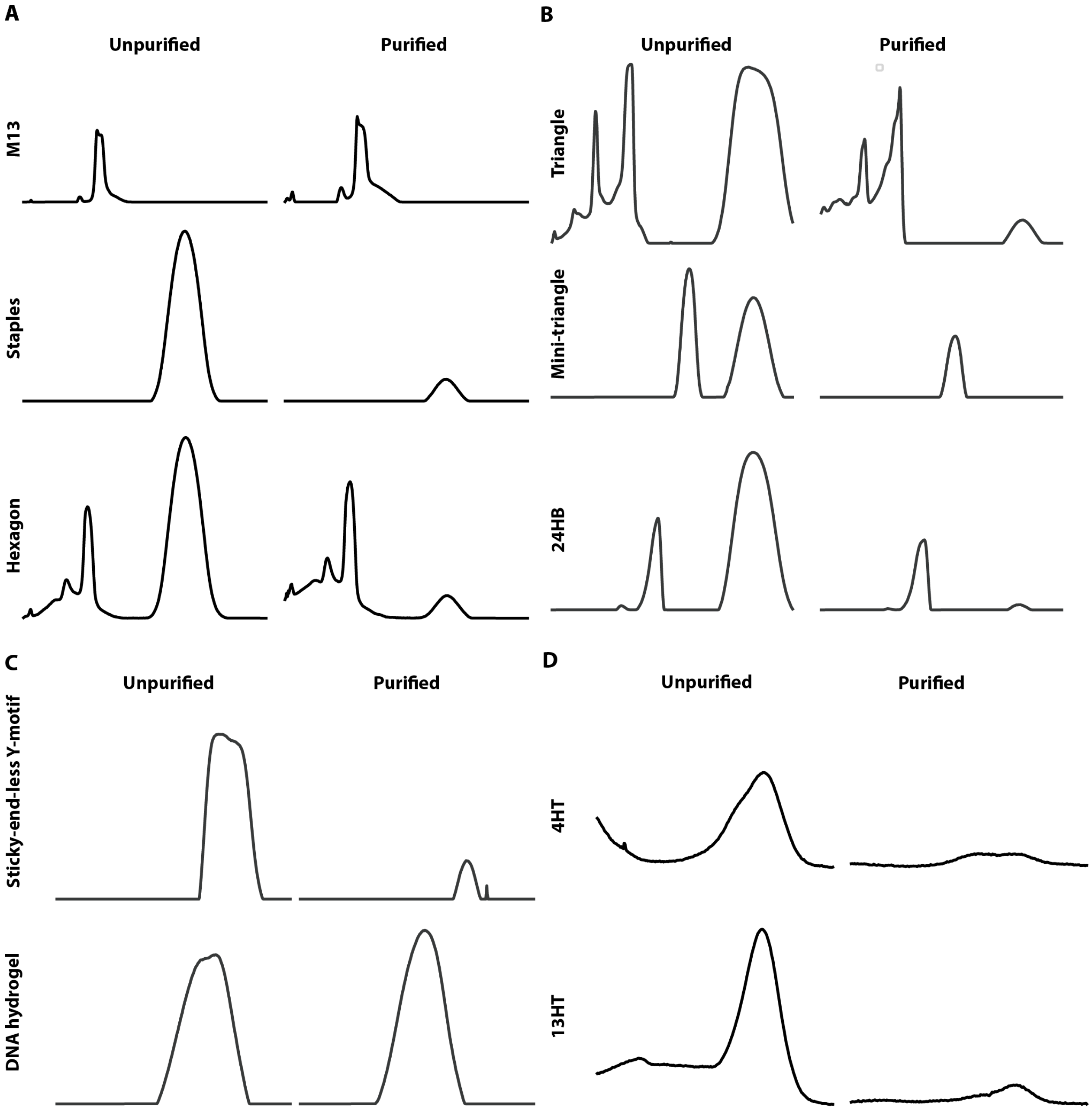

Supplementary Figure S7. Normalized intensity profiles of agarose electrophoresis of DNA self-assembled structures before and after purification by W/W-droplet fractionation. **A**. Hexagonal DNA origami and its components. **B.** DNA origami with different scaffolds. **C**. Motif-based structures. **D**. DNA microtubes.

4. SUPPLEMENTARY MATERIAL REFERENCES

[1] D. Ishikawa, Y. Suzuki, C. Kurokawa, M. Ohara, M. Tsuchiya, M. Morita, M. Yanagisawa, M. Endo, R. Kawano, M. Takinoue, *Angew. Chem. Int. Ed.* **2019**, *131*, 15443.

[2] Y. Sato, T. Sakamoto, M. Takinoue, *Sci. Adv.* **2020**, *6*, 1.

[3] P. W. K. Rothemund, *Nature* **2006**, *440*, 297.

[4] H. Said, V. J. Schüller, F. J. Eber, C. Wege, T. Liedl, C. Richert, *Nanoscale* **2013**, *5*, 284.

[5] A. Kuzyk, R. Schreiber, Z. Fan, G. Pardatscher, E. M. Roller, A. Högele, F. C. Simmel, A. O. Govorov, T. Liedl, *Nature* **2012**, *483*, 311.

[6] A. M. Maier, W. Bae, D. Schi, J. F. Emmerig, M. Schi, T. Liedl, *ACS Nano* **2017**, *11*, 1301.

[7] P. Yin, R. F. Hariadi, S. Sahu, H. M. T. Choi, H. P. Sung, T. H. LaBean, J. H. Reif, *Science* **2008**, *321*, 824.

[8] T. des Coudres, *Organ* **1898**, *7*, 225.

[9] P.-A. Albertsson, *Partition of Cell Particles and Macromolecules*, Wiley-Interscience, New York, **1971**.

[10] A. A. Louis, P. G. Bolhuis, E. J. Meijer, J. P. Hansen, *J. Chem. Phys.* **2002**, *117*, 1893.

[11] Z. Zhao, Q. Li, X. Ji, R. Dimova, R. Lipowsky, Y. Liu, *J. Chromatogr. A* **2016**, *1452*, 107.

[12] N. Zęibacz, S. A. Wieczorek, T. Kalwarczyk, M. Fiałkowski, R. Hołyst, *Soft Matter* **2011**, *7*, 7181.

[13] P. Aimar, M. Meireles, V. Sanchez, *J. Membr. Sci.* **1990**, *54*, 321.

[14] R. Bourne, R. Bourne, *Fundamentals of Digital Imaging in Medicine* **2010**, *9*, 185.

[15] C. A. Schneider, W. S. Rasband, K. W. Eliceiri, *Nat. Methods* **2012**, *9*, 671.
